# The Temporal Relationship between Depressive Symptoms and Loneliness: The Moderating Role of Self-Compassion

**DOI:** 10.3390/bs13060472

**Published:** 2023-06-05

**Authors:** Shujian Wang, Qihui Tang, Yichao Lv, Yanqiang Tao, Xiangping Liu, Liang Zhang, Gang Liu

**Affiliations:** 1 Faculty of Psychology, Beijing Normal University, Beijing 100875, China; 202121061093@mail.bnu.edu.cn (S.W.); 202221061033@mail.bnu.edu.cn (Q.T.); 202231061033@mail.bnu.edu.cn (Y.L.); 202131061049@mail.bnu.edu.cn (Y.T.); 89034@bnu.edu.cn (X.L.); 2 Beijing Key Laboratory of Applied Experimental Psychology, National Demonstration Center for Experimental Psychology Education, Beijing 100875, China; 3 College Students’ Mental Health Education Center, Northeast Agricultural University, Harbin 150030, China; zhangliangpsy@neau.edu.cn; 4 Department of Psychiatry, Affiliated Nanjing Brain Hospital, Nanjing Medical University, Nanjing 210029, China

**Keywords:** depression, loneliness, self-compassion, comorbidity, network analysis

## Abstract

Loneliness and depression are significant mental health challenges among college students; however, the intricate relationship between these phenomena remains unclear, particularly in the context of self-compassion. In this comprehensive study, we employ a cross-lagged panel network (CLPN) analysis to investigate the symptom-level association between depression and loneliness while exploring the potential moderating influence of self-compassion. Our sample consisted of 2785 college students, who were categorized into high- and low-self-compassion groups based on scores from the Self-Compassion Scale. Depressive symptoms were assessed using the Patient Health Questionnaire-9, while the UCLA Loneliness Scale-8 measured loneliness expressions. Our findings indicate that self-compassion plays a crucial role in the relationship between depression and loneliness. Specifically, we observed distinctive patterns within the high and low-self-compassion groups. In the low-self-compassion group, “energy” emerged as the most influential symptom, whereas “motor function” exhibited the highest influence in the high-self-compassion group. Furthermore, among individuals with high self-compassion, the pathway from depression to loneliness was characterized by “guilt—being alone when desired,” while the reverse path from loneliness to depression encompassed “left out—feeling sad” and “left out—anhedonia.” Conversely, in the low-self-compassion group, depression and loneliness demonstrated a more intricate mutual triggering relationship, suggesting that self-compassion effectively moderates the association between these variables. This study provides valuable insights into the underlying mechanisms driving the interplay between depression and loneliness, shedding light on the pivotal role of self-compassion in this intricate dynamic.

## 1. Introduction

In modern society, depression has become an increasingly common mental disorder, posing serious harm to both sufferers and the entire community [1]. This issue is particularly severe among the college student population. According to the 2022 annual report released by the Center for Collegiate Mental Health, de-identified data contributed by 180 college and university counseling centers revealed that they received over 1.2 million appointments for psychological treatment in the 2021 academic year alone [2]. The most recent meta-analysis indicates that the global prevalence of depression among college students has reached 33.6% (95% confidence interval 29.3–37.8%) and confirms a strong association between loneliness and the severity of depression [3].

Loneliness is defined as the unpleasant subjective perception caused by poor interpersonal relationships [4]. Numerous studies have demonstrated a substantial correlation between depression and loneliness [5,6,7]. However, there is considerable debate regarding how these two distinct psychological problems are linked, as well as the causal relationship between depression and loneliness [8,9,10,11]. Moreover, some researchers have suggested that the comorbidity of different mental issues should be considered at the level of specific manifestations, rather than simply adding up total scores across different items in the scale [12]. Therefore, one aim of the current study was to analyze the relationship between depression and loneliness in terms of concrete observable performances using longitudinal data from a network analysis perspective. Meanwhile, given the critical contribution of self-compassion to mental health, we investigate whether self-compassion can alleviate the relationship between depression and loneliness.

### 1.1. The Bidirectional Relationship between Loneliness and Depression among the College Student Population

Humans are social animals by nature, and the need for a sense of belonging is a fundamental psychological need that drives us to form and maintain positive interpersonal relationships [13,14]. Although the strength and methods of satisfying this need may vary from person to person [15], individuals may experience feelings of loneliness when a discrepancy exists between their desired and actual social connections [16]. When entering college, adolescents tend to develop higher expectations for their social relationships [17,18] and become more concerned about their social status [19]. This stage of life is also characterized by adolescents seeking independence and separating from their parents and primary families [20], which typically results in a decrease in interpersonal support from family members [21]. Therefore, if young students have not developed the necessary skills to cope with the changing social environment or have unrealistic expectations regarding social relationships, they may experience loneliness [4]. A meta-analysis has shown that college students experience more severe problems from loneliness than the general adult population [22].

Depression is a mental disorder characterized by persistent sadness, hopelessness, a loss of interest in previously enjoyable activities, and other symptoms [23]. Given that depression is a more global and heterogeneous disorder that involves appraisals across multiple domains and poses greater threats to both individuals and societies [24], some researchers suggest from a preventative standpoint that loneliness may be a contributing factor in the onset of depression [22]. For instance, a longitudinal study found that loneliness at the start of a semester can predict depression six weeks later [7]. Similarly, Rotenberg and Hymel demonstrated that loneliness during mid-adolescence can predict an increase in depression after two and a half years [19]. Meanwhile, individuals with a sense of belonging to a group were found to be less likely to experience depression two and four years later [25]. Erzen and Çikrikci argued that loneliness could lead adolescents to believe that no one understands them and that no one can help them when needed, which can trigger depression [22].

On the other hand, some researchers have suggested that loneliness may also be conceptualized as an outcome of depression [26]. Slavin and Rainer found that girls who experienced depressive symptoms tended to become more socially isolated within their families [27]. Additionally, people are more reluctant to spend time with individuals who are depressed compared to non-depressed individuals and the general population, as it can lead to them experiencing more negative emotions themselves [10]. Joiner and Metalsky conducted a three-week naturalistic study on college roommates and found that depressed students reported engaging in more negative feedback seeking, and their depression level could predict an increase in rejection by their roommates [28]. Furthermore, some case studies have indicated that social withdrawal can occur exclusively during major depressive episodes, leading to impaired maintenance of existing social relationships and difficulty establishing new ones, thus contributing to an increased sense of loneliness [29].

Several other studies have taken an integrative approach that considers both causal paths, but the triggering relationship between depression and loneliness remains unclear [9,30]. This uncertainty may be due to the fact that these studies have adopted the traditional view of depression and loneliness as latent variables, which ignores the heterogeneity of different intuitive and tangible manifestations and the interrelations between them [31]. Hence, the traditional perspective often oversimplifies the complexity of mental health issues [12].

The cross-lagged panel network (CLPN) is a novel approach that offers insights into how psychopathological dimensions interact over time at the symptom level. This approach analyzes how observations at one time point predict observations at the next, revealing transdiagnostic mechanisms and identifying symptoms that play a predictive or influential role in the network [32]. Moreover, the CLPN can detect significant symptom interactions that may indicate potential causal relationships and points of intervention to disrupt negative processes [33]. Therefore, the current study attempts to explore the comorbidity mechanisms of loneliness and depression at a more granular level using the CLPN approach.

### 1.2. The Moderating Role of Self-Compassion

Self-compassion is a concept rooted in Eastern Buddhism, which involves treating oneself with kindness, understanding, and support, especially in the face of challenging emotions. This practice of self-care can help individuals cope with difficulties and ultimately facilitate even the most difficult changes [34]. Neff further explored self-compassion from a social psychological perspective and conceptualized the construct into three components: mindfulness, which entails being aware of the present moment experience in a balanced way rather than overidentifying with it; self-kindness, which involves taking a kind psychological stance towards oneself rather than self-judgment; and common humanity, which involves recognizing that all human beings experience failures and setbacks rather than feeling isolated by one’s failures [35].

A substantial body of research has consistently demonstrated the potential of self-compassion to serve as a protective factor that promotes emotional resilience [34,36,37]. Individuals who possess self-compassion are less inclined to engage in harsh self-judgment and self-criticism when confronted with negative life experiences, such as failure or alienation. Instead, they tend to demonstrate kindness, gentleness, and consideration towards themselves while recognizing their feelings of loneliness as a shared human experience [38,39]. These thoughts of common humanity contribute to a sense of connection with the broader human community and partially fulfill the need for social relatedness. Furthermore, a systematic review revealed that self-compassion enhances emotion regulation in response to social threats [40]. Through self-compassion training, individuals develop improved abilities to regulate their emotions, thereby preventing typical defensive and anxiety-driven responses to negative social events [41]. Some researchers also found that self-compassion can alleviate the adverse effects of rumination on interpersonal problems and depressive symptoms [42,43,44]. Liu et al. argued that when individuals encounter interpersonal isolation and frustration, self-compassion enables them to treat themselves kindly, acknowledge that others may also experience difficulties in their social interactions, and maintain a balanced perspective that regards solitude as an objective state [45]. These factors collectively contribute to decreased sensitivity to loneliness. Importantly, self-compassion’s distinct buffering effect on loneliness may prevent its generalization to other mental health issues [46].

On the other hand, self-compassion has been found to be beneficial in reducing the negative effects of depressive symptoms. For instance, Kuyken et al. demonstrated that increasing self-compassion through mindfulness-based cognitive therapy led to a significant reduction in depressive symptoms during follow-up assessments [47]. Additionally, self-compassion has been identified as a protective psychological factor against depression [48]. A meta-analysis further supported the importance of self-compassion as an explanatory variable for mental health and resilience [36]. Notably, a positive psychology study showed that engaging in self-compassion exercises helped individuals reduce depressive emotions and negative rumination, leading to improved mood, increased optimism, and enhanced well-being even six months later [49]. Considering the role of optimism in fostering positive interpersonal interactions, self-compassion is likely to assist individuals with depression in rebuilding healthy relationships [50]. Moreover, self-compassion can enhance an individual’s positive attitude towards others, thereby promoting higher social motivation and encouraging participation in social activities, ultimately alleviating feelings of loneliness. Consequently, self-compassion’s protective effect on the social functioning of individuals with depression may help prevent social isolation and alleviate loneliness.

Given the potential impact of self-compassion on the association between depressive symptoms and loneliness, it is imperative to explore this relationship longitudinally in individuals with varying levels of self-compassion. By investigating both high- and low-self-compassion groups, we aim to examine the potential differences in the longitudinal dynamics between depressive symptoms and loneliness, thereby shedding light on how self-compassion may moderate this relationship. This investigation will yield valuable insights into the protective role of self-compassion and its implications for mental health outcomes.

### 1.3. The Current Study

In recent years, there has been a growing body of research that examines loneliness not merely as a unidimensional construct but also explores the impact of specific manifestations of loneliness on mental health issues in a more detailed manner. For instance, von Känel et al. employed a network approach to compare the association between loneliness and depressive symptoms at both the symptom and syndrome levels [51]. Similarly, Owczarek et al. investigated the relationship between the three components of loneliness (“lack of companionship,” “feeling lonely,” and “feeling left out”) and anxiety symptoms [52]. Additionally, Ramos-Vera et al. argued that although some items in the UCLA Loneliness Scale have similar content, they actually measure distinct aspects [53]. For example, “feeling isolated” may reflect a sense of disconnection from others, while “feeling lonely” refers to an emotional state of sadness resulting from a lack of interpersonal relationships.

Building on these previous studies, our research aims to address several limitations in the existing literature regarding the association between depression and loneliness and examine the moderating effect of self-compassion. By employing the CLPN method to analyze data at a more granular level and utilizing a longitudinal design, our study allows for possible causal inferences regarding the directionality of the relationship between depression and loneliness, as well as the potential protective role of self-compassion. Given the research findings presented above, we assume that

**Hypothesis** **1** **(H1):** *Students in the low-self-compassion group have a denser depression–loneliness network structure than those in the high-self-compassion group.*

**Hypothesis** **2** **(H2):** *Self-compassion plays a moderation role in the relationship between loneliness and depression, manifesting that the transmission mechanism between depression and loneliness is more straightforward in the high-self-compassion group compared to the low-self-compassion group.*

Additionally, this study was conducted during the COVID-19 pandemic. Given that previous studies have shown that quarantine during COVID-19 can exacerbate individuals’ loneliness and depression [53,54], we collected data from participants at two time points, the first wave during initial quarantine and the second wave after release (three months later), to explore the effect of the quarantine policies on these processes. We assume that:

**Hypothesis** **3** **(H3):** *After three months of quarantine, students have a stronger and denser depression–loneliness network structure.*

The findings of the current study may have important implications for developing interventions to alleviate the negative effects of loneliness and depression, particularly during times of emergency social exclusion policies.

## 2. Method

### 2.1. Participants and Procedure

We collected data from 6710 college students at [redacted for peer review] university in Harbin, China, on 26 September 2021 (the first wave) and 3731 students from the same school on 27 December 2021 (the second wave). After merging the datasets based on students’ school numbers, we recruited 2785 participants (58.6% females, *mean*_age_ = 18.34, *SD*_age_ = 0.92, range 18 to 28) for our study. Before participating in the assessment, students and their parents provided signed informed consent forms. All participants completed the questionnaires via the Wenjuanxing online questionnaire platform (https://www.wjx.cn/, accessed on 31 May 2023).

To examine the moderating effect of self-compassion (SC), we divided participants into two groups based on their self-compassion scale (SCS) ratings. Our sample’s mean SCS score was 36.71, with a standard deviation of 9.42. Participants with SCS scores in the top 27% were placed in the high-SC group, while those with SCS scores in the bottom 27% were placed in the low-SC group. After sifting through the data, the final sample consisted of 1502 students, with 750 in the high-SC group (60.2% females, *mean* _age_ = 18.33, *SD*_age_ = 0.94) and 752 in the low-SC group (50.5% females, *mean*_age_ = 18.39, *SD*_age_ =0.91).

This study was reviewed and approved by the ethical committee at [redacted for peer review] University (Reference number: 202112220084).

### 2.2. Measures

#### 2.2.1. Self-Compassion Scale (SCS)

The Self-Compassion Scale (SCS), is a widely used measure for assessing levels of self-compassion [55]. The scale assesses thoughts, emotions, and behaviors associated with various components of self-compassion, including self-kindness, mindfulness, and common humanity. Participants rate their responses to 12 Likert-style questions on a scale ranging from 1 (“almost never”) to 5 (“almost always”). The Chinese version of the SCS was revised by Chen et al. [56] and has been shown to be both valid and reliable [57]. In our study, the SCS demonstrated good internal consistency, with a Cronbach’s *α* value of 0.87.

#### 2.2.2. Patient Health Questionnaire-9 (PHQ-9)

The Patient Health Questionnaire-9 (PHQ-9), comprises nine items that assess depressive symptoms, including anhedonia, sad mood, sleep, energy, appetite, guilt, concentration, motor function, and suicide ideation in the last two weeks [23]. Each item is graded on a scale ranging from 0 (“not at all”) to 3 (“nearly every day”), with higher scores indicating higher levels of depression severity. The Chinese version of the PHQ-9 has been shown to have good reliability and validity [58,59]. In our study, the PHQ-9 showed good internal consistency with a Cronbach’s *α* value of 0.90.

#### 2.2.3. UCLA Loneliness Scale-8 (ULS-8)

Hays and DiMatteo developed the ULS-8 based on the ULS-4 and ULS-20 and verified its reliability and applicability [60]. The ULS-8 is a self-report questionnaire composed of eight 5-point Likert-style items scored from 1 (“never”) to 4 (“always”), with items 3 (“I am an outgoing person”) and 6 (“I can find companionship when I want it”) being reverse scored. The Chinese version of the ULS-8 was revised by Wu and Yao [61]. Higher scores on the ULS-8 indicate a higher level of loneliness. In our current study, the ULS-8 had good internal consistency, with a Cronbach’s *α* value of 0.88.

### 2.3. Network Analysis

The present research employed extended Bayesian Information Criterion (EBIC) and graphical least absolute shrinkage and selection operator (LASSO) network models [62] to establish four contemporaneous network structures. The R packages *bootnet 1.4.3* [63] and *qgraph 1.6.9* [64] were used for network estimation and visualization. Additionally, we used the *glmnet* package [65] to conduct two cross-lagged panel networks (CLPN) to examine the relationships between the first and second assessments over time. The detailed statistical procedures are available in the Supplementary Materials.

## 3. Result

### 3.1. Item Check and Descriptive Statistics

The means, standard deviations, skewness, kurtosis, and *t*-test results of all items in the two groups are shown in Table 1 and Figure S1. The item check results showed that no items should be excluded, indicating that the PHQ-9 and ULS-8 meet the requirements for conducting network analysis. Most scores in the low-SC group were higher than in the high-SC group at first- and second-time points. The *t*-test results for data from two time points are shown in Table S1.

### 3.2. Contemporaneous Network Structures

**Figure 1:**
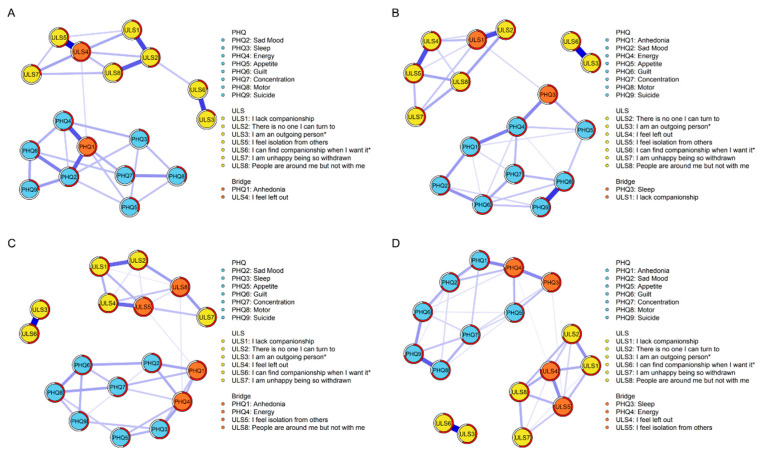
Network structures. (**A**) High-SC group at the first time point. (**B**) High-SC group at the second time point. (**C**) Low-SC group at the first time point. (**D**) Low-SC group at the second time point.

**Figure 2:**
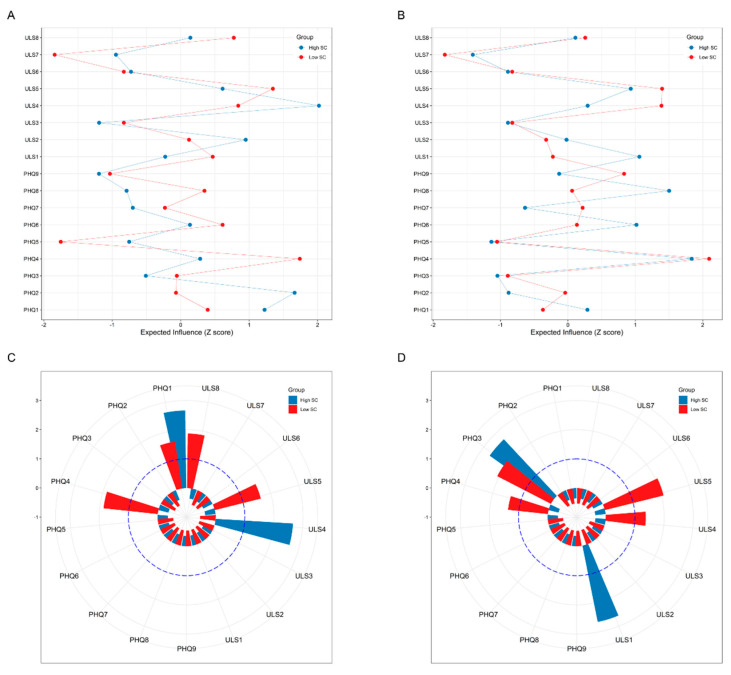
Network centrality indices. (**A**) *EI* values of the low-SC and high-SC groups at the first time point. (**B**) *EI* values of the low-SC and high-SC groups at the second time point. (**C**) Bridge values of the low-SC and high-SC groups at the first time point. (**D**) Bridge values of the low-SC and high-SC groups at the second time point.

Four depression–loneliness contemporaneous networks (groups (Low-SC vs. High-SC) * time-points (First wave vs. Second wave)) are shown in Figure 1. All weighted adjacency matrices are shown in Tables S2–S5. Centrality *EI* and bridge values are presented in Figure 2.

For the high-SC group at the first time point (Figure 1, Part A), the edge of “left out”—“isolation” (ULS4-ULS5) showed the strongest association; for the other three networks (Figure 1, Parts B, C, and D), the edge of “introverted”—“being alone when desired” (ULS3-ULS6) showed the strongest association. Regarding symptom centrality, for the high-SC group at the first time point, “left out” (ULS4) had the strongest node *EI*; for the low-SC group at the first time point, “energy” (PHQ4) had the strongest node *EI* (see Figure 2, Part A). “Energy” (PHQ4) was also the most influential node in both high-SC and low-SC groups at the second time point (see Figure 2, Part B).

According to the criteria proposed by Sánchez Hernández [66], the bridge symptoms at the first time point were “anhedonia” (PHQ1) and “left out” (ULS4) in the high-SC group and “anhedonia” (PHQ1), “energy” (PHQ4), “isolation” (ULS5), and “lonely in a crowd” (ULS8) in the low-SC group (see Figure 2, Part C). At the second time point, the bridge symptoms were “sleep” (PHQ3) and “lack companionship” (ULS1) in the high-SC group and “sleep” (PHQ3), “energy” (PHQ4), “left out” (ULS4), and “isolation” (ULS5) in the low-SC group (see Figure 2, Part D).

### 3.3. Network Comparison Test

The network comparison test results are shown in Table 2 and Figures S2 and S3.

Regarding cross-sectional NCTs, the global strength of the low-SC group in the first wave was significantly higher than that of the high-SC group (Strength _high-SC_ = 7.28, Strength _low-SC_ = 7. 95, *S* = 0.67, *p* = 0.002). There was also a significant difference in the edge distribution between the two groups in the first wave. For longitudinal NCTs, the global strength of the high-SC group in the second wave was significantly higher than in the first wave (Strength _T1_ = 7.28, Strength _T2_ = 7. 82, *S* = 0.54, *p* = 0.006). The network edge distributions of high- and low-SC groups between the two time points also exhibited significant variation.

### 3.4. Temporal Network Structures

**Figure 3:**
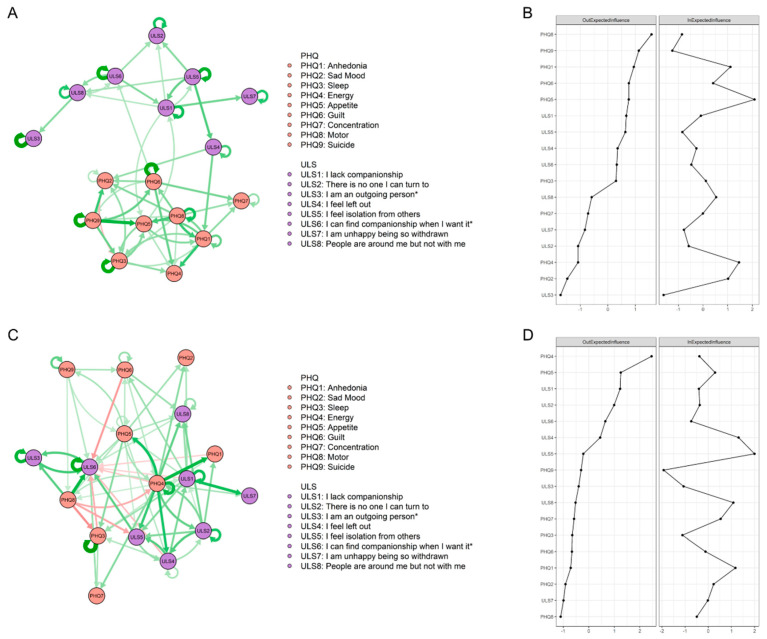
CLPN structures and centrality indexes. (**A**) CLPN of high-SC group. (**B**) *OEI* and *IEI* values of high-SC group. (**C**) CLPN of low-SC group. (**D**) *OEI* and *IEI* values of low-SC group.

The CLPN structures are visualized in Figure 3. All edge weights are shown in LASSO cross-lagged regression matrixes in Tables S6 and S7. The autoregression path values are shown in Figure S4.

For the high-SC group, 156 (54%) edges were not zero among 289 possible edges. Between two symptom clusters, only seven symptoms were found to be interrelated: “guilt” (PHQ6) was able to positively predict “being alone when desired” (ULS6); “left out” (ULS4) was able to positively predict “anhedonia” (PHQ1) and “sad mood” (PHQ2); “lack companionship” (ULS1) and “appetite” (PHQ5) were able to predict each other, as shown in part A of Figure 3. Part B in Figure 3 showed the *OEI* and *IEI* values: “motor” (PHQ8) had the highest node *OEI*, followed by “suicide” (PHQ9) and “anhedonia” (PHQ1). “Appetite” (PHQ5) had the highest node *IEI*, followed by “energy” (PHQ4) and “anhedonia” (PHQ1).

For the low-SC group, 160 (55%) edges were not zero among 289 possible edges. The interconnection between the two symptom clusters was much stronger, with all symptoms showing some degree of association with the other community. Except for autoregression paths, the edge of “energy”—“anhedonia” (PHQ4-PHQ1) showed the strongest cross-lagged association, followed by the edge of “lack companionship”—“unhappy due to withdrawal” (ULS1-ULS7) and the edge of “energy”—“appetite” (PHQ4-PHQ5), shown in part C of Figure 3. In part D of Figure 3, “energy” (PHQ4) had the highest node *OEI*, followed by “appetite” (PHQ5) and “lack companionship” (ULS1). “Isolation” (ULS5) had the highest node *IEI*, followed by “left out” (ULS4) and “anhedonia” (PHQ1).

### 3.5. Network Accuracy and Stability

The case-dropping results are shown in Figure S5, and all *CS-Cs* are shown in Table 3. Case-dropping test results indicated good stability for all centrality indicators.

Approximately 95% of bootstrapped *CI*s of edges were narrow (see Figure S6), suggesting that edges were trustworthy. The nonparametric bootstrap procedure results revealed that most comparisons among edge weights and centrality indicators were statistically significant (Figures S7–S9).

### 3.6. Unidimensional Loneliness Network Structures

**Figure 4:**
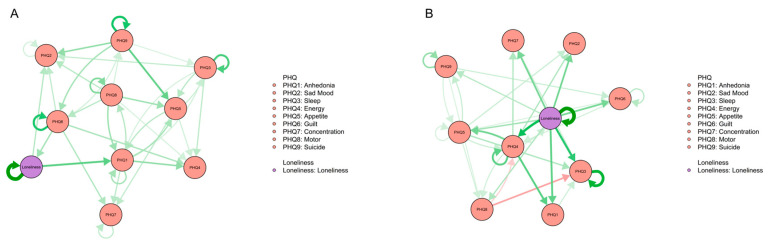
The unidimensional loneliness CLPN structures. (**A**) CLPN of high-SC group. (**B**) CLPN of low-SC group.

To test the rationality and reliability of analyzing loneliness at the item level, we considered loneliness as a unidimensional variable in the CLPN analysis to examine the differences between the structure of the unidimensional loneliness network and the multidimensional loneliness network. The unidimensional loneliness cross-lagged networks are shown in Figure 4. All edge weights are presented in LASSO cross-lagged regression matrixes in Tables S8 and S9.

When loneliness is taken as a unidimensional variable, the structure of the network is broadly consistent with that of the original network. In the high-SC group, the mechanism of transmission from loneliness to depression was “loneliness—anhedonia (PHQ1)” and “loneliness—sad mood (PHQ2)”. The mechanism of transmission from depression to loneliness consisted of “guilt (PHQ6)—loneliness”. In the low-SC group, the network structure was more complex. Every depressive symptom was connected to loneliness, which was consistent with the multidimensional loneliness cross-lagged network analysis.

## 4. Discussion

The present study examined the correlation and cross-lagged effects between specific symptoms of depression and loneliness, while also investigating the moderating effect of self-compassion in this process. Hypotheses 1 and 2 have been confirmed, while hypothesis 3 has been partially validated. Specifically, only students in the high-self-compassion group experienced a significant negative impact from the isolation measures. Several noteworthy results emerged from the study.

### 4.1. The Contemporaneous Symptom Network Structures and t-Test Results between High- and Low-Self-Compassion Groups at Two Time Points

In terms of symptom centrality, “energy” was found to be the most influential symptom, exhibiting stability across time and groups. Specifically, “energy” had the highest EI value at both time points for low-self-compassion individuals and at the second time point for high-self-compassion individuals. Low energy reflects an individual’s tiredness at both the cognitive and behavioral levels [23]. A meta-analysis showed that tiredness was particularly severe in depressed populations [67]. Early psychiatry also viewed depression as a disorder characterized by retardation of thoughts and movements, a perspective that some modern researchers still hold [68]. Studies using physiological monitoring devices have found that depressed patients exhibit less motor activity than the general population, and successful treatment with antidepressants often leads to a reduction in motor retardation [69,70,71]. This finding may suggest that screening for individual depression risk may be more effective at the behavioral level than the emotional level, particularly for large populations such as college students. This is because, on the one hand, the current findings align with established views, suggesting that the core problem of depression can also be represented by behavioral issues [72]. On the other hand, depressed patients may conceal their emotional problems due to social stigmatization, whereas behavioral indicators are easier to observe and document [73]. This finding also highlights the potential benefits of exercise programs in improving the mental health of students experiencing depression and loneliness. A substantial body of research has confirmed the detrimental effects of a lack of exercise on mental health [74]. However, it is important to note that many sports activities on university campuses are typically conducted in group settings. This poses a challenge for lonely students, as they may have fewer opportunities to engage in exercise, thereby increasing their likelihood of falling into a detrimental cycle characterized by a lack of physical activity, easily feeling tired, and exacerbating depressive symptoms [75,76]. Meanwhile, intervention studies have shown that physical activity can help reduce feelings of loneliness [77]. Given these findings, it would be beneficial for universities to consider actively encouraging students, particularly those experiencing loneliness, to participate in group exercise programs. By doing so, universities can alleviate feelings of loneliness and help prevent the onset or worsening of depression among students.

A noteworthy finding of our study is that both the network density and item level of the high-self-compassion group significantly increased after three months of quarantine, while no significant change was observed in the low-self-compassion group. In the network approach, although item-level severity and network density may exhibit correlation, they fundamentally capture distinct aspects of mental health issues. The former focuses on the overall severity of external manifestations, while the latter examines the intricacies of internal processes [78]. These results suggest that individuals with high levels of self-compassion may be more susceptible to the impact of forced quarantine, and long-term isolation measures can pose both external and internal threats to their mental health.

A possible explanation for these findings is the presence of a ceiling effect in the low-self-compassion group. The increase in network density is believed to be the outcome of repeated symptom triggering [78]. During the COVID-19 quarantine, the government implemented restrictions on public gatherings and private outings. The *t*-test results from the first wave between the two groups indicated that the level of loneliness was higher in the low-self-compassion group compared to the high-self-compassion group, suggesting that students with low self-compassion may have had lower levels of social engagement prior to the quarantine, aligning with previous studies [79,80]. Therefore, it is plausible that only high-self-compassion students were more significantly affected by the quarantine measures. For students with low self-compassion, the chronic negative events in their daily lives may have solidified the connection between depression and loneliness to a point where it becomes less susceptible to further deepening. The fact that the network density of the low-self-compassion group was higher than that of the high-self-compassion group in the first wave supports this assumption.

A study conducted in the UK during the pandemic lockdown also came to a similar conclusion [53]. There have also been numerous studies demonstrating that individuals with low self-compassion suffer from much lower levels of mental health and are more depleted by injury and illness than those with high self-compassion [38,81,82]. This suggests that psychological treatments for individuals with low self-compassion will be more difficult. It is important to consider these findings when designing interventions for individuals struggling with loneliness and depression, particularly during periods of long-term isolation, such as pandemics or prolonged lockdowns. Clinicians may need to tailor their approaches based on an individual’s level of self-compassion to be most effective.

### 4.2. Cross-Lagged Panel Networks between High- and Low-Self-Compassion Groups

The CLPN analysis results illustrate how individuals’ loneliness and depression can be mutually reinforcing. Hypothesis 2 was effectively tested, providing support for the notion that the transmission mechanism between depression and loneliness is more simple in the high-self-compassion group compared to the low-self-compassion group.

For individuals who have high levels of self-compassion, their depression and loneliness problems appear to be bidirectional when loneliness is considered a unidimensional variable. Furthermore, at the symptom level, the relationship between these two clusters becomes more apparent: “guilt” in the first wave positively predicted “being alone when desired” in the second wave, while “left out” in the first wave positively predicted “sad mood” and “anhedonia” in the second wave.

Guilt is a powerful and aversive self-conscious emotion that often makes individuals feel a sense of shame or embarrassment about something they have done or failed to do [83]. As a moral-social emotion, guilt is always experienced when an individual relates to others and is based on mutual evaluations and judgments of the self and others [84]. Therefore, the concept of “others” and the connection with others are the core subjects of guilt [85]. Individuals with high levels of guilt perceive themselves as immutable and worthless to others and therefore consider relationships to be meaningless and unchangeable [86]. This may cause them to develop social withdrawal and struggle to communicate with others honestly because they may avoid social interactions due to fear of being judged or rejected [87]. This impairment in social functioning may leave them unaccompanied when they face challenging events and require social support.

Feeling left out may arise when individuals perceive that they are not included in a particular social group or activity that they desire to be a part of. This feeling is often considered to be the result of social exclusion or rejection and can have many negative mental consequences [88]. Twenge et al. found that social exclusion decreases prosocial behavior and can lead to negative emotions [89]. This finding was supported by the social baseline theory, which posits that social relationships are crucial for emotional regulation [90]. When individuals are excluded from a social group, they may experience a loss of social support and emotional regulation, leading to feelings of sadness and distress [91]. Moreover, anhedonia is the loss of pleasure or interest in previously enjoyable activities [92]. As the amotivation dimension of negative symptoms of psychotic disorders, anhedonia has been considered a motivational and cognitive consequence of social exclusion [93]. In summary, depression appears to spread throughout the loneliness network primarily through the “guilty—being alone when desired” pathway for individuals with high self-compassion, while loneliness triggers feelings of depression through the “left out—sad mood” and “left out—anhedonia” pathways.

In the low-self-compassion group, the relationship between depression and loneliness becomes even more complex. Almost every symptom was connected to at least one symptom in another cluster. CLPN results visualized this phenomenon clearly: in the high-self-compassion group’s network structure, the two symptom clusters were divided into two distinct communities, while the nodes from two different symptom clusters in the low-self-compassion group were intertwined. This finding provided further evidence to support our research hypothesis, which posits that self-compassion can act as a buffer between depression and loneliness. Numerous studies have found that a higher level of self-compassion is associated with improved psychological constructs, including social connectedness and psychopathology [36,94]. Self-compassion can help individuals maintain a more interconnected self-concept and view themselves as part of the larger human experience, thus reducing the impact of negative experiences and emotions [95]. Bloch also suggested that individuals with higher levels of self-compassion feel less threatened and anxious and dwell less on negative interpersonal interactions [96].

Conversely, people with low self-compassion are more susceptible to interpersonal frustration and negative emotions [97]. For these individuals, triggering any single symptom may cause fluctuations in the entire depression–loneliness network, leading to a negative spiral. Although high self-compassion is closely associated with a safe, supportive upbringing in early childhood [35,98], later interventions to increase self-compassion are still possible. Compassionate mind training (CMT), imagery building, the gestalt two-chair technique, mindfulness-based stress reduction (MBSR), dialectical behavior therapy (DBT), and acceptance and commitment therapy (ACT) have all been proven to improve self-compassion to some extent [94]. Clinicians may consider these modalities when treating depressed patients with high levels of loneliness and social relational deficits.

### 4.3. Limitations

The study has several limitations that need to be acknowledged. Firstly, the current study did not compare variations in network structures between depressed and non-depressed groups. Future studies may test the effects of self-compassion on individuals with different levels of depression, which could contribute to a better clarification of the current findings. Secondly, the study used a non-clinical sample of college students, which may not be representative of clinical individuals with depressive disorder. Therefore, caution should be exercised when generalizing the results to clinical populations. Thirdly, although the study used longitudinal data, the relationships between symptoms, especially causality, should be treated cautiously. Future experimental studies are needed to validate the current conclusions.

## 5. Conclusions

This study is the first to investigate the relationship between depression and loneliness and the moderating role of self-compassion from a network analysis perspective. We found that feelings of loneliness and depression are less likely to spread in people with a high level of self-compassion, and we revealed the mechanism underlying the mutual transmission of loneliness and depression. The current study contributes to our understanding of the protective role of self-compassion for mental health and has important implications for designing interventions to help individuals struggling with loneliness and depression, particularly during periods of long-term isolation. Further research is needed to address the limitations and advance our understanding of these relationships.

## Figures and Tables

**Table 1:** Descriptive information and *t*-test results of two groups.

		Low-SC Group (N = 752)				High-SC Group (N = 750)				*t*-Test	
		*Mean*	*SD*	*Skew*	*Kurtosis*	*Mean*	*SD*	*Skew*	*Kurtosis*	*p*	*Cohen’s d*
T1	PHQ1	1.54	0.76	1.51	2.05	1.36	0.62	1.76	3.05	<0.001	0.25
	PHQ2	1.46	0.69	1.62	2.76	1.31	0.58	1.87	3.15	<0.001	0.23
	PHQ3	1.36	0.71	2.15	4.14	1.28	0.56	2.13	4.49	0.017	0.12
	PHQ4	1.53	0.71	1.33	1.66	1.35	0.60	1.66	2.52	<0.001	0.27
	PHQ5	1.43	0.71	1.79	3.05	1.33	0.62	2.01	4.05	0.006	0.14
	PHQ6	1.37	0.68	1.98	3.80	1.25	0.55	2.38	5.75	<0.001	0.21
	PHQ7	1.49	0.74	1.57	2.05	1.35	0.66	1.98	3.57	<0.001	0.20
	PHQ8	1.19	0.51	3.23	11.57	1.22	0.54	2.75	7.86	0.249	−0.06
	PHQ9	1.08	0.35	5.68	36.84	1.13	0.42	3.91	16.90	0.015	−0.13
	ULS1	2.19	0.95	0.12	−1.12	1.72	0.87	0.76	−0.79	<0.001	0.52
	ULS2	1.93	0.90	0.47	−0.92	1.67	0.84	0.85	−0.56	<0.001	0.30
	ULS3	0.60	0.78	1.29	1.27	1.18	1.18	0.51	−1.25	<0.001	−0.58
	ULS4	2.14	0.87	0.12	−0.99	1.69	0.85	0.75	−0.93	<0.001	0.53
	ULS5	2.21	0.90	0.05	−1.01	1.74	0.86	0.62	−1.10	<0.001	0.54
	ULS6	0.74	0.86	1.02	0.32	1.32	1.17	0.35	−1.35	<0.001	−0.56
	ULS7	2.01	0.92	0.42	−0.89	1.74	0.87	0.76	−0.65	<0.001	0.30
	ULS8	1.85	0.89	0.70	−0.48	1.63	0.81	0.87	−0.64	<0.001	0.26
T2	PHQ1	1.53	0.76	1.52	2.02	1.37	0.68	1.95	3.48	<0.001	0.22
	PHQ2	1.39	0.63	1.78	3.42	1.30	0.59	2.09	4.28	<0.001	0.14
	PHQ3	1.48	0.78	1.73	2.46	1.40	0.77	2.04	3.51	0.017	0.10
	PHQ4	1.56	0.77	1.45	1.93	1.38	0.67	1.89	3.43	<0.001	0.25
	PHQ5	1.43	0.77	1.88	2.84	1.32	0.64	2.17	4.51	0.006	0.15
	PHQ6	1.34	0.67	2.18	4.66	1.26	0.59	2.54	6.58	<0.001	0.13
	PHQ7	1.37	0.69	1.92	3.23	1.28	0.60	2.25	4.69	<0.001	0.15
	PHQ8	1.21	0.55	2.94	9.07	1.20	0.52	2.98	9.63	0.249	0.02
	PHQ9	1.13	0.47	4.07	17.85	1.16	0.48	3.45	12.93	0.015	−0.06
	ULS1	2.01	0.94	0.33	−1.12	1.77	0.87	0.61	−1.04	<0.001	0.27
	ULS2	1.89	0.89	0.49	−0.93	1.70	0.85	0.76	−0.77	<0.001	0.21
	ULS3	0.90	0.99	0.91	−0.22	1.15	1.16	0.53	−1.20	<0.001	−0.23
	ULS4	2.01	0.90	0.29	−1.12	1.77	0.87	0.61	−1.04	<0.001	0.27
	ULS5	2.08	0.91	0.14	−1.23	1.77	0.86	0.55	−1.17	<0.001	0.34
	ULS6	0.96	1.00	0.75	−0.55	1.26	1.17	0.38	−1.35	<0.001	−0.28
	ULS7	2.05	0.94	0.37	−0.97	1.82	0.92	0.69	−0.76	<0.001	0.25
	ULS8	1.84	0.87	0.61	−0.73	1.68	0.84	0.81	−0.68	<0.001	0.19

**Table 2:** Network comparison results (1000 permutations).

	Edge Invariance	Global Invariance
High-SC—low-SC (first time)	*M* = 0.35	*S* = 0.67
	*p* < 0.001	*p* = 0.002
High-SC—low-SC (second time)	*M* = 0.17	*S* = 0.22
	*p* = 0.51	*p* = 0.16
High-SC (longitudinal)	*M* = 0.38	*S* = 0.54
	*p* < 0.001	*p* = 0.006
Low-SC (longitudinal)	*M* = 0.26	*S* = 0.08
	*p* = 0.03	*p* = 0.57

Note. *M*: The value of the maximum difference in edge weights. *S*: The value of difference in sum of all edge weights.

**Table 3:** The *CS-Cs* of all *EI* values.

	*EI* (First Time)	*EI* (Second Time)	*OEI*	*IEI*
High-SC group	0.595	0.439	0.263	0.407
Low-SC group	0.439	0.594	0.364	0.379

## Data Availability

The data presented in this study are available on request from the corresponding author.

## Supplementary Materials

The following supporting information can be downloaded at: https://www.mdpi.com/article/10.3390/bs13060472/s1.
