# Supplementary material for: The Temporal Relationship between Depressive Symptoms and Loneliness: The Moderating Role of Self-Compassion
Source: Behav Sci (Basel). 2023 Jun 5;13(6):472. doi: 10.3390/bs13060472 (PMC10294791; doi:10.3390/bs13060472)
Supplement: Supplementary file 1 [file behavsci-13-00472-s001.zip › behavsci-2418918-supplementary/behavsci-2418918-supplementary_for proofreading.pdf]

Network analysis method

Item check

The Goldbricker method was used to test the heterogeneity between various items. If more than 75% of the correlation coefficients between two items and all other items were not significantly different, these two items were considered to measure the same manifestation, and one of them should be excluded. The item check process was conducted by the R package *networktools 1.5.0* (P. Jones, 2022).

Contemporaneous network estimation and indices

All data analyses were conducted in *R version 4.2.2* (R Core Team, 2022). The symptom networks were estimated by the extended Bayesian information criterion (EBIC) graphical least absolute shrinkage and selection operator (LASSO) method (Epskamp & Fried, 2018). The correlation matrix was shrunk to obtain a sparser and easier network structure. Each node in the network represents a symptom, and each edge represents the adjusted correlation coefficient between two nodes. Line thickness represents the strength of association. The red and blue edges represent negative and positive correlations, respectively. The R packages *bootnet 1.4.3* (Epskamp, Waldorp, et al., 2018) and *qgraph 1.6.9* (Epskamp et al., 2012) were used for network estimation and visualization.

The expected influence (*EI*) was used to characterize the node's centrality. The *EI* of a node is calculated by adding up the values of all edges connected to this node, which represents the importance and influence of this symptom in the network (Robinaugh et al., 2016). The R package *qgraph 1.6.9* was used to calculate *EI* values (Epskamp et al., 2012). We also calculated each node's predictability (i.e., R^2) using the R package *mgm 1.2-12* (Haslbeck & Waldorp, 2020).

Bridge symptoms represent the channel linking different symptom communities (P. J. Jones et al., 2021). Referring to the previous research (Sánchez Hernández et al., 2021), we chose bridge symptoms based on the criterion of standardized values of bridge strength ≥ 1 in the present study.

Network comparison

The network comparison test (NCT) was used to assess edge invariance (distributions of edge weights between two networks) and global invariance (sum of all edge weights between two networks). NCTs were conducted by the R package *NetworkComparisonTest 2.2.1* (van Borkulo et al., 2022).

Cross-lagged panel network estimation and indices

The cross-lagged panel network (CLPN) was conducted to explore the interrelationships between different depressive symptoms and loneliness behaviors by using the *glmnet* package (Friedman et al., 2010). As in the cross-sectional networks, we applied the LASSO method to shrink the small regression path to 0 and chose the best-penalized model with a 10-fold cross-validation method (Schlechter et al., 2022). The CLPN can depict how nodes (i.e., specific symptoms) at the first time point predict nodes at the second time point after adjusting all other variables at the first time point (Epskamp, 2020). Each edge in the CLPN signifies a directed regression path: the edges of each node pointing to itself signify the autoregression coefficients, and the edges pointing to other nodes signify the cross-lagged regression coefficients. The thickness and color depth of the edge represent the strength of this regression path. The green and red arrows indicate positive and negative effects, respectively.

For directed CLPNs, there are two centrality indices: in-expected influence (*IEI*) and out-expected influence (*OEI*). *OEI* is calculated by summing up all values of outgoing edges connected to the symptom, which signifies the extent to which one symptom predicts the others. *IEI* is calculated by summing up all values of incoming edges connected to the symptom, representing the extent to which the others predict one symptom.

Network accuracy and stability

The accuracy and stability of the networks were tested using three bootstrapping approaches implemented by the R package *bootnet 1.4.3* (Epskamp et al., 2018). First, we calculated all edge weights' 95% bootstrapped (1000 iterations) confidence intervals

(*CI*s). The narrower *CI*s indicated a more accurate network. Second, we used the case-dropping procedure to estimate the stability of centrality indices, which can provide the correlation stability coefficient (*CS*-*C*). Generally, *CS*-*C* should be ≥ 0.25, preferably ≥ 0.5 (Costenbader & Valente, 2003). Third, we estimated the 95% bootstrapped (1000 iterations) *CI*s of differences between edge weights and centrality indices to test whether these differences were statistically significant.

References

Costenbader, E., & Valente, T. W. (2003). The stability of centrality measures when networks are sampled. *Social Networks*, *25*(4), 283–307. https://doi.org/10.1016/S0378-8733(03)00012-1

Epskamp, S. (2020). Psychometric network models from time-series and panel data. *Psychometrika*, *85*(1), 206–231. https://doi.org/10.1007/s11336-020-09697-3

Epskamp, S., Borsboom, D., & Fried, E. I. (2018). Estimating psychological networks and their accuracy: A tutorial paper. *Behavior Research Methods*, *50*(1), 195–212. https://doi.org/10.3758/s13428-017-0862-1

Epskamp, S., Cramer, A. O. J., Waldorp, L. J., Schmittmann, V. D., & Borsboom, D. (2012). qgraph: Network Visualizations of Relationships in Psychometric Data. *Journal of Statistical Software*, *48*(4), 1–18. https://doi.org/10.18637/jss.v048.i04

Epskamp, S., & Fried, E. I. (2018). A tutorial on regularized partial correlation networks. *Psychological Methods*, *23*(4), 617–634. https://doi.org/10.1037/met0000167

Epskamp, S., Waldorp, L. J., Mõttus, R., & Borsboom, D. (2018). The Gaussian Graphical Model in Cross-Sectional and Time-Series Data. *Multivariate Behavioral Research*, *53*(4), 453–480. https://doi.org/10.1080/00273171.2018.1454823

Friedman, J. H., Hastie, T., & Tibshirani, R. (2010). Regularization Paths for Generalized Linear Models via Coordinate Descent. *Journal of Statistical Software*, *33*(1), 1–22. https://doi.org/10.18637/jss.v033.i01

Haslbeck, J. M. B., & Waldorp, L. J. (2020). mgm: Estimating Time-Varying Mixed Graphical Models in High-Dimensional Data. *Journal of Statistical Software*, *93*(8), 1–46. https://doi.org/10.18637/jss.v093.i08

Jones, P. (2022). *networktools: Tools for Identifying Important Nodes in Networks*. https://CRAN.R-project.org/package=networktools

Jones, P. J., Ma, R., & McNally, R. J. (2021). Bridge Centrality: A Network Approach to Understanding Comorbidity. *Multivariate Behavioral Research*, *56*(2), 353–367. https://doi.org/10.1080/00273171.2019.1614898

R Core Team. (2022). *R: A Language and Environment for Statistical Computing*. https://www.R-project.org/

Robinaugh, D. J., Millner, A. J., & McNally, R. J. (2016). Identifying highly influential nodes in the complicated grief network. *Journal of Abnormal Psychology*, *125*(6), 747–757. https://doi.org/10.1037/abn0000181

Sánchez Hernández, M. O., Carrasco, M. A., & Holgado-Tello, F. P. (2021). Anxiety and Depression Symptoms in Spanish Children and Adolescents: An Exploration of Comorbidity from the Network Perspective. *Child Psychiatry & Human Development*. https://doi.org/10.1007/s10578-021-01286-4

Schlechter, P., Hellmann, J. H., McNally, R. J., & Morina, N. (2022). The longitudinal course of posttraumatic stress disorder symptoms in war survivors: Insights from cross-lagged panel network analyses. *Journal of Traumatic Stress*, *35*(3), 879–890. https://doi.org/10.1002/jts.22795

van Borkulo, C. D., van Bork, R., Boschloo, L., Kossakowski, J. J., Tio, P., Schoevers, R. A., Borsboom, D., & Waldorp, L. J. (2022). Comparing network structures on three aspects: A permutation test. *Psychological Methods*. https://doi.org/10.1037/met0000476.

Table S1. The t-test results for data from two time points.

	High-SC group (N = 750)			Low-SC group (N = 752)		
	**T1**	**T2**	***p***	**T1**	**T2**	***p***
**PHQ1**	1.54 (0.76)	1.53 (0.76)	0.812	1.36 (0.62)	1.37 (0.68)	0.812
**PHQ2**	1.46 (0.69)	1.39 (0.63)	0.031	1.31 (0.58)	1.30 (0.59)	0.725
**PHQ3**	1.36 (0.71)	1.48 (0.78)	0.002	1.28 (0.56)	1.40 (0.77)	<0.001
**PHQ4**	1.53 (0.71)	1.56 (0.77)	0.420	1.35 (0.60)	1.38 (0.67)	0.395
**PHQ5**	1.43 (0.71)	1.43 (0.77)	1.000	1.33 (0.62)	1.32 (0.64)	0.653
**PHQ6**	1.37 (0.68)	1.34 (0.67)	0.282	1.25 (0.55)	1.26 (0.59)	0.716
**PHQ7**	1.49 (0.74)	1.37 (0.69)	0.002	1.35 (0.66)	1.28 (0.60)	0.034
**PHQ8**	1.19 (0.51)	1.21 (0.55)	0.408	1.22 (0.54)	1.20 (0.52)	0.436
**PHQ9**	1.08 (0.35)	1.13 (0.47)	0.007	1.12 (0.42)	1.16 (0.48)	0.137
**ULS1**	2.19 (0.95)	2.01 (0.94)	<0.001	1.72 (0.87)	1.77 (0.87)	0.262
**ULS2**	1.93 (0.90)	1.89 (0.89)	0.356	1.67 (0.84)	1.70 (0.85)	0.464
**ULS3**	0.60 (0.78)	0.90 (0.99)	<0.001	1.18 (1.18)	1.15 (1.16)	0.644
**ULS4**	2.14 (0.87)	2.01 (0.90)	0.003	1.69 (0.85)	1.77 (0.87)	0.068
**ULS5**	2.21 (0.90)	2.08 (0.91)	0.003	1.74 (0.86)	1.77 (0.86)	0.473
**ULS6**	0.74 (0.86)	0.96 (1.00)	<0.001	1.32 (1.17)	1.26 (1.17)	0.321
**ULS7**	2.01 (0.92)	2.05 (0.94)	0.439	1.74 (0.87)	1.82 (0.92)	0.096
**ULS8**	1.85 (0.89)	1.84 (0.87)	0.883	1.63 (0.81)	1.68 (0.84)	0.222

Table S2. Weighted adjacency matrix of PHQ and ULS symptoms (high-SC group at the first time).

	PHQ1	PHQ2	PHQ3	PHQ4	PHQ5	PHQ6	PHQ7	PHQ8	PHQ9	ULS1	ULS2	ULS3	ULS4	ULS5	ULS6	ULS7	ULS8
PHQ1	0.000	0.300	0.000	0.325	0.119	0.000	0.155	0.000	0.000	0.000	0.000	0.000	0.073	0.000	0.000	0.000	0.000
PHQ2	0.300	0.000	0.124	0.104	0.114	0.246	0.000	0.000	0.193	0.000	0.000	0.000	0.000	0.000	0.000	0.000	0.000
PHQ3	0.000	0.124	0.000	0.154	0.122	0.000	0.000	0.137	0.000	0.000	0.000	0.000	0.000	0.000	0.000	0.000	0.000
PHQ4	0.325	0.104	0.154	0.000	0.000	0.154	0.000	0.000	0.000	0.000	0.000	0.000	0.000	0.000	0.000	0.000	0.000
PHQ5	0.119	0.114	0.122	0.000	0.000	0.000	0.000	0.121	0.000	0.000	0.000	0.000	0.000	0.000	0.000	0.000	0.000
PHQ6	0.000	0.246	0.000	0.154	0.000	0.000	0.126	0.000	0.173	0.000	0.000	0.000	0.000	0.000	0.000	0.000	0.000
PHQ7	0.155	0.000	0.000	0.000	0.000	0.126	0.000	0.209	0.000	0.000	0.000	0.000	0.000	0.000	0.000	0.000	0.000
PHQ8	0.000	0.000	0.137	0.000	0.121	0.000	0.209	0.000	0.000	0.000	0.000	0.000	0.000	0.000	0.000	0.000	0.000
PHQ9	0.000	0.193	0.000	0.000	0.000	0.173	0.000	0.000	0.000	0.000	0.000	0.000	0.000	0.000	0.000	0.000	0.000
ULS1	0.000	0.000	0.000	0.000	0.000	0.000	0.000	0.000	0.000	0.000	0.346	0.000	0.167	0.096	0.000	0.000	0.000
ULS2	0.000	0.000	0.000	0.000	0.000	0.000	0.000	0.000	0.000	0.346	0.000	0.000	0.133	0.000	0.116	0.000	0.307
ULS3	0.000	0.000	0.000	0.000	0.000	0.000	0.000	0.000	0.000	0.000	0.000	0.000	0.000	0.000	0.367	0.000	0.000
ULS4	0.073	0.000	0.000	0.000	0.000	0.000	0.000	0.000	0.000	0.167	0.133	0.000	0.000	0.484	0.000	0.179	0.134
ULS5	0.000	0.000	0.000	0.000	0.000	0.000	0.000	0.000	0.000	0.096	0.000	0.000	0.484	0.000	0.000	0.113	0.124
ULS6	0.000	0.000	0.000	0.000	0.000	0.000	0.000	0.000	0.000	0.000	0.116	0.367	0.000	0.000	0.000	0.000	0.000
ULS7	0.000	0.000	0.000	0.000	0.000	0.000	0.000	0.000	0.000	0.000	0.000	0.000	0.179	0.113	0.000	0.000	0.136
ULS8	0.000	0.000	0.000	0.000	0.000	0.000	0.000	0.000	0.000	0.000	0.307	0.000	0.134	0.124	0.000	0.136	0.000

Table S3. Weighted adjacency matrix of PHQ and ULS symptoms (high-SC group at the second time).

	PHQ1	PHQ2	PHQ3	PHQ4	PHQ5	PHQ6	PHQ7	PHQ8	PHQ9	ULS1	ULS2	ULS3	ULS4	ULS5	ULS6	ULS7	ULS8
PHQ1	0.000	0.265	0.000	0.336	0.098	0.076	0.103	0.000	0.000	0.000	0.000	0.000	0.000	0.000	0.000	0.000	0.000
PHQ2	0.265	0.000	0.000	0.000	0.000	0.282	0.000	0.000	0.121	0.000	0.000	0.000	0.000	0.000	0.000	0.000	0.000
PHQ3	0.000	0.000	0.000	0.315	0.217	0.000	0.000	0.000	0.000	0.106	0.000	0.000	0.000	0.000	0.000	0.000	0.000
PHQ4	0.336	0.000	0.315	0.000	0.149	0.105	0.183	0.068	0.000	0.000	0.000	0.000	0.000	0.000	0.000	0.000	0.000
PHQ5	0.098	0.000	0.217	0.149	0.000	0.000	0.000	0.158	0.000	0.000	0.000	0.000	0.000	0.000	0.000	0.000	0.000
PHQ6	0.076	0.282	0.000	0.105	0.000	0.000	0.229	0.154	0.163	0.000	0.000	0.000	0.000	0.000	0.000	0.000	0.000
PHQ7	0.103	0.000	0.000	0.183	0.000	0.229	0.000	0.196	0.000	0.000	0.000	0.000	0.000	0.000	0.000	0.000	0.000
PHQ8	0.000	0.000	0.000	0.068	0.158	0.154	0.196	0.000	0.519	0.000	0.000	0.000	0.000	0.000	0.000	0.000	0.000
PHQ9	0.000	0.121	0.000	0.000	0.000	0.163	0.000	0.519	0.000	0.000	0.000	0.000	0.000	0.000	0.000	0.000	0.000
ULS1	0.000	0.000	0.106	0.000	0.000	0.000	0.000	0.000	0.000	0.000	0.466	0.000	0.183	0.106	0.000	0.156	0.000
ULS2	0.000	0.000	0.000	0.000	0.000	0.000	0.000	0.000	0.000	0.466	0.000	0.000	0.111	0.000	0.000	0.000	0.246
ULS3	0.000	0.000	0.000	0.000	0.000	0.000	0.000	0.000	0.000	0.000	0.000	0.000	0.000	0.000	0.666	0.000	0.000
ULS4	0.000	0.000	0.000	0.000	0.000	0.000	0.000	0.000	0.000	0.183	0.111	0.000	0.000	0.459	0.000	0.000	0.125
ULS5	0.000	0.000	0.000	0.000	0.000	0.000	0.000	0.000	0.000	0.106	0.000	0.000	0.459	0.000	0.000	0.185	0.244
ULS6	0.000	0.000	0.000	0.000	0.000	0.000	0.000	0.000	0.000	0.000	0.000	0.666	0.000	0.000	0.000	0.000	0.000
ULS7	0.000	0.000	0.000	0.000	0.000	0.000	0.000	0.000	0.000	0.156	0.000	0.000	0.000	0.185	0.000	0.000	0.231
ULS8	0.000	0.000	0.000	0.000	0.000	0.000	0.000	0.000	0.000	0.000	0.246	0.000	0.125	0.244	0.000	0.231	0.000

Table S4. Weighted adjacency matrix of PHQ and ULS symptoms (low-SC group at the first time).

	PHQ1	PHQ2	PHQ3	PHQ4	PHQ5	PHQ6	PHQ7	PHQ8	PHQ9	ULS1	ULS2	ULS3	ULS4	ULS5	ULS6	ULS7	ULS8
PHQ1	0.000	0.254	0.172	0.203	0.000	0.000	0.203	0.000	0.000	0.000	0.000	0.000	0.000	0.071	0.000	0.000	0.000
PHQ2	0.254	0.000	0.000	0.240	0.000	0.247	0.000	0.000	0.087	0.000	0.000	0.000	0.000	0.000	0.000	0.000	0.000
PHQ3	0.172	0.000	0.000	0.258	0.219	0.000	0.000	0.000	0.181	0.000	0.000	0.000	0.000	0.000	0.000	0.000	0.000
PHQ4	0.203	0.240	0.258	0.000	0.184	0.000	0.152	0.000	0.000	0.000	0.000	0.000	0.000	0.000	0.000	0.000	0.079
PHQ5	0.000	0.000	0.219	0.184	0.000	0.000	0.000	0.155	0.000	0.000	0.000	0.000	0.000	0.000	0.000	0.000	0.000
PHQ6	0.000	0.247	0.000	0.000	0.000	0.000	0.187	0.288	0.215	0.000	0.000	0.000	0.000	0.000	0.000	0.000	0.000
PHQ7	0.203	0.000	0.000	0.152	0.000	0.187	0.000	0.260	0.000	0.000	0.000	0.000	0.000	0.000	0.000	0.000	0.000
PHQ8	0.000	0.000	0.000	0.000	0.155	0.288	0.260	0.000	0.191	0.000	0.000	0.000	0.000	0.000	0.000	0.000	0.000
PHQ9	0.000	0.087	0.181	0.000	0.000	0.215	0.000	0.191	0.000	0.000	0.000	0.000	0.000	0.000	0.000	0.000	0.000
ULS1	0.000	0.000	0.000	0.000	0.000	0.000	0.000	0.000	0.000	0.000	0.420	0.000	0.225	0.211	0.000	0.000	0.058
ULS2	0.000	0.000	0.000	0.000	0.000	0.000	0.000	0.000	0.000	0.420	0.000	0.000	0.082	0.084	0.000	0.000	0.273
ULS3	0.000	0.000	0.000	0.000	0.000	0.000	0.000	0.000	0.000	0.000	0.000	0.000	0.000	0.000	0.706	0.000	0.000
ULS4	0.000	0.000	0.000	0.000	0.000	0.000	0.000	0.000	0.000	0.225	0.082	0.000	0.000	0.413	0.000	0.081	0.173
ULS5	0.071	0.000	0.000	0.000	0.000	0.000	0.000	0.000	0.000	0.211	0.084	0.000	0.413	0.000	0.000	0.179	0.096
ULS6	0.000	0.000	0.000	0.000	0.000	0.000	0.000	0.000	0.000	0.000	0.000	0.706	0.000	0.000	0.000	0.000	0.000
ULS7	0.000	0.000	0.000	0.000	0.000	0.000	0.000	0.000	0.000	0.000	0.000	0.000	0.081	0.179	0.000	0.000	0.284
ULS8	0.000	0.000	0.000	0.079	0.000	0.000	0.000	0.000	0.000	0.058	0.273	0.000	0.173	0.096	0.000	0.284	0.000

Table S5. Weighted adjacency matrix of PHQ and ULS symptoms (low-SC group at the second time).

	PHQ1	PHQ2	PHQ3	PHQ4	PHQ5	PHQ6	PHQ7	PHQ8	PHQ9	ULS1	ULS2	ULS3	ULS4	ULS5	ULS6	ULS7	ULS8
PHQ1	0.000	0.250	0.000	0.344	0.000	0.064	0.148	0.000	0.000	0.000	0.000	0.000	0.000	0.000	0.000	0.000	0.000
PHQ2	0.250	0.000	0.000	0.193	0.000	0.237	0.000	0.113	0.070	0.000	0.000	0.000	0.000	0.000	0.000	0.000	0.000
PHQ3	0.000	0.000	0.000	0.341	0.191	0.000	0.116	0.000	0.000	0.000	0.000	0.000	0.000	0.067	0.000	0.000	0.000
PHQ4	0.344	0.193	0.341	0.000	0.218	0.090	0.000	0.000	0.000	0.000	0.000	0.000	0.049	0.000	0.000	0.000	0.000
PHQ5	0.000	0.000	0.191	0.218	0.000	0.081	0.121	0.077	0.000	0.000	0.000	0.000	0.000	0.000	0.000	0.000	0.000
PHQ6	0.064	0.237	0.000	0.090	0.081	0.000	0.122	0.000	0.300	0.000	0.000	0.000	0.000	0.000	0.000	0.000	0.000
PHQ7	0.148	0.000	0.116	0.000	0.121	0.122	0.000	0.224	0.178	0.000	0.000	0.000	0.000	0.000	0.000	0.000	0.000
PHQ8	0.000	0.113	0.000	0.000	0.077	0.000	0.224	0.000	0.468	0.000	0.000	0.000	0.000	0.000	0.000	0.000	0.000
PHQ9	0.000	0.070	0.000	0.000	0.000	0.300	0.178	0.468	0.000	0.000	0.000	0.000	0.000	0.000	0.000	0.000	0.000
ULS1	0.000	0.000	0.000	0.000	0.000	0.000	0.000	0.000	0.000	0.000	0.319	0.000	0.239	0.206	0.000	0.069	0.000
ULS2	0.000	0.000	0.000	0.000	0.000	0.000	0.000	0.000	0.000	0.319	0.000	0.000	0.163	0.125	0.000	0.000	0.208
ULS3	0.000	0.000	0.000	0.000	0.000	0.000	0.000	0.000	0.000	0.000	0.000	0.000	0.000	0.000	0.727	0.000	0.000
ULS4	0.000	0.000	0.000	0.049	0.000	0.000	0.000	0.000	0.000	0.239	0.163	0.000	0.000	0.332	0.000	0.084	0.246
ULS5	0.000	0.000	0.067	0.000	0.000	0.000	0.000	0.000	0.000	0.206	0.125	0.000	0.332	0.000	0.000	0.161	0.224
ULS6	0.000	0.000	0.000	0.000	0.000	0.000	0.000	0.000	0.000	0.000	0.000	0.727	0.000	0.000	0.000	0.000	0.000
ULS7	0.000	0.000	0.000	0.000	0.000	0.000	0.000	0.000	0.000	0.069	0.000	0.000	0.084	0.161	0.000	0.000	0.238
ULS8	0.000	0.000	0.000	0.000	0.000	0.000	0.000	0.000	0.000	0.000	0.208	0.000	0.246	0.224	0.000	0.238	0.000

Table S6. LASSO cross-lagged regression matrix for high-SC group. Each number in the matrix represents the regression coefficient of the symptom in the same row on its left side (first time point) predicting the symptom in the same column on its upper side (second time point).

	PHQ1	PHQ2	PHQ3	PHQ4	PHQ5	PHQ6	PHQ7	PHQ8	PHQ9	ULS1	ULS2	ULS3	ULS4	ULS5	ULS6	ULS7	ULS8
PHQ1	0.123	0.000	0.093	0.146	0.118	0.048	0.094	0.107	0.028	0.007	0.000	0.000	0.000	0.000	0.000	0.000	0.000
PHQ2	0.023	0.068	-0.071	0.000	0.000	0.000	0.000	0.000	0.000	0.000	0.000	0.000	0.036	0.032	0.000	0.003	0.000
PHQ3	0.070	0.089	0.231	0.096	0.102	0.000	0.000	0.019	0.008	0.012	0.000	0.007	0.000	0.000	0.029	0.000	0.043
PHQ4	0.000	0.000	0.000	0.026	0.000	0.000	0.010	0.000	0.000	0.000	0.000	0.057	0.000	0.000	0.057	0.000	0.000
PHQ5	0.100	0.020	0.095	0.069	0.051	0.033	0.088	0.041	0.023	0.067	0.042	0.000	0.015	0.000	0.000	0.000	0.000
PHQ6	0.000	0.094	0.000	0.120	0.000	0.248	0.112	0.055	0.069	0.000	0.000	0.000	0.000	0.000	0.070	0.020	0.054
PHQ7	0.046	0.000	0.000	0.000	0.035	0.000	0.090	0.000	0.000	0.022	0.000	0.000	0.036	0.039	0.000	0.000	0.039
PHQ8	0.142	0.102	0.067	0.064	0.139	0.108	0.000	0.168	0.068	0.000	0.000	0.000	0.000	0.000	0.056	0.050	0.011
PHQ9	0.000	0.143	0.120	0.000	0.196	0.123	0.000	0.045	0.247	0.000	0.000	0.000	0.004	0.056	0.000	0.000	0.000
ULS1	0.022	0.000	0.055	0.011	0.065	0.000	0.057	0.007	0.005	0.173	0.065	0.004	0.030	0.071	0.000	0.114	0.064
ULS2	0.009	0.000	0.000	0.000	0.000	0.000	0.000	0.000	0.000	0.008	0.165	0.000	0.019	0.036	0.000	0.000	0.052
ULS3	0.000	0.000	-0.057	0.000	0.000	0.000	0.000	0.000	0.000	0.000	0.000	0.274	0.000	0.000	0.000	0.018	0.000
ULS4	0.113	0.089	0.058	0.051	0.024	0.045	0.000	0.005	0.022	0.015	0.000	0.000	0.137	0.038	0.000	0.029	0.000
ULS5	0.016	0.000	0.012	0.027	0.002	0.016	0.032	0.000	0.000	0.122	0.099	0.000	0.134	0.211	0.016	0.009	0.078
ULS6	0.005	0.003	0.059	0.000	0.000	0.044	0.006	0.001	0.002	0.102	0.082	0.011	0.029	0.012	0.228	0.031	0.095
ULS7	0.006	0.000	-0.017	0.000	0.000	0.000	0.000	0.000	0.000	0.032	0.030	0.000	0.058	0.000	0.040	0.150	0.037
ULS8	0.000	0.000	0.000	0.016	0.007	0.040	0.000	0.000	0.002	0.000	0.000	0.100	0.000	0.000	0.065	0.018	0.152

Table S7. LASSO cross-lagged regression matrix for low-SC group. Each number in the matrix represents the regression coefficient of the symptom in the same row on its left side (first time point) predicting the symptom in the same column on its upper side (second time point).

	PHQ1	PHQ2	PHQ3	PHQ4	PHQ5	PHQ6	PHQ7	PHQ8	PHQ9	ULS1	ULS2	ULS3	ULS4	ULS5	ULS6	ULS7	ULS8
PHQ1	0.010	0.008	0.068	0.009	0.030	0.000	0.024	0.000	0.000	0.016	0.000	0.000	0.000	0.000	-0.064	0.000	0.000
PHQ2	0.000	0.041	0.000	0.000	0.000	0.000	0.024	0.000	0.000	0.000	0.000	0.000	0.000	0.000	0.000	0.000	-0.029
PHQ3	0.020	0.000	0.330	0.036	0.028	0.042	0.083	0.016	0.000	0.000	0.000	0.000	0.000	0.000	-0.105	0.000	0.000
PHQ4	0.227	0.118	0.046	0.227	0.195	0.034	0.121	0.080	0.035	0.062	0.148	0.000	0.187	0.172	-0.076	0.088	0.149
PHQ5	0.059	0.097	0.119	0.092	0.078	0.074	0.023	0.078	0.092	0.050	0.018	0.000	0.077	0.062	0.089	0.000	0.085
PHQ6	0.000	0.016	0.000	0.076	0.000	0.107	0.000	0.000	0.000	0.000	0.000	0.000	0.000	0.035	-0.130	0.014	0.103
PHQ7	0.031	0.000	0.000	0.000	0.000	0.000	0.004	0.021	0.000	0.000	0.000	0.000	0.000	0.000	0.100	0.000	0.000
PHQ8	0.000	0.000	-0.146	-0.109	0.000	0.000	0.000	0.000	0.000	0.000	0.000	0.155	-0.047	-0.116	0.228	0.000	-0.059
PHQ9	0.000	0.024	0.000	0.000	0.068	0.088	0.031	0.062	0.150	0.000	0.000	0.000	-0.031	0.000	0.081	0.000	-0.039
ULS1	0.003	0.089	0.101	0.027	0.080	0.102	0.016	0.061	0.053	0.205	0.047	0.000	0.054	0.123	-0.062	0.205	0.106
ULS2	0.122	0.020	0.011	0.127	0.000	0.025	0.059	0.011	0.025	0.060	0.188	0.000	0.160	0.125	-0.001	0.014	0.134
ULS3	0.000	0.004	0.000	0.045	0.000	0.000	0.018	0.000	0.003	0.000	0.000	0.246	0.000	0.000	0.168	0.000	0.000
ULS4	0.037	0.054	0.081	0.052	0.000	0.000	0.043	0.000	0.000	0.127	0.093	0.000	0.092	0.158	-0.085	0.049	0.025
ULS5	0.000	0.000	0.000	0.000	0.000	0.000	0.000	0.000	0.000	0.000	0.000	0.009	0.109	0.023	0.151	0.053	0.001
ULS6	0.036	0.045	0.041	0.033	0.052	0.050	0.034	0.048	0.038	0.025	0.031	0.160	0.039	0.046	0.267	0.000	0.052
ULS7	0.000	-0.028	0.000	0.000	0.000	0.000	0.000	0.000	-0.002	0.000	0.000	0.000	0.000	0.000	-0.013	0.001	0.000
ULS8	0.000	0.000	0.000	0.000	0.000	0.000	0.000	0.000	0.000	0.046	0.053	0.000	0.000	0.008	0.072	0.000	0.110

Table S8. LASSO cross-lagged regression matrix of the unidimensional loneliness network structure for low-SC group.

	PHQ1	PHQ2	PHQ3	PHQ4	PHQ5	PHQ6	PHQ7	PHQ8	PHQ9	Loneliness
PHQ1	0.016	0.006	0.079	0.011	0.016	0.000	0.022	0.000	0.000	0.000
PHQ2	0.000	0.044	0.000	0.000	0.000	0.000	0.022	0.000	0.000	0.000
PHQ3	0.025	0.000	0.342	0.040	0.027	0.044	0.082	0.013	0.000	0.000
PHQ4	0.213	0.116	0.047	0.213	0.191	0.024	0.114	0.074	0.031	0.079
PHQ5	0.052	0.084	0.118	0.080	0.067	0.063	0.016	0.070	0.086	0.045
PHQ6	0.000	0.012	0.000	0.071	0.000	0.107	0.000	0.000	0.003	0.002
PHQ7	0.035	0.000	0.000	0.000	0.000	0.000	0.001	0.017	0.000	0.005
PHQ8	0.000	0.000	-0.169	-0.092	0.000	0.000	0.000	0.000	0.000	0.000
PHQ9	0.000	0.005	0.000	0.000	0.067	0.082	0.033	0.064	0.152	0.000
Loneliness	0.209	0.186	0.246	0.285	0.136	0.185	0.184	0.115	0.106	0.459

Table S9. LASSO cross-lagged regression matrix of the unidimensional loneliness network structure for high-SC group.

	PHQ1	PHQ2	PHQ3	PHQ4	PHQ5	PHQ6	PHQ7	PHQ8	PHQ9	Loneliness
PHQ1	0.129	0.002	0.072	0.152	0.124	0.049	0.101	0.108	0.030	0.001
PHQ2	0.020	0.069	0.000	0.000	0.000	0.000	0.000	0.000	0.000	0.000
PHQ3	0.069	0.092	0.222	0.100	0.105	0.000	0.000	0.021	0.010	0.018
PHQ4	0.000	0.000	0.000	0.029	0.000	0.000	0.011	0.000	0.000	0.000
PHQ5	0.105	0.027	0.080	0.074	0.056	0.032	0.096	0.044	0.027	0.023
PHQ6	0.000	0.096	0.000	0.122	0.000	0.249	0.115	0.055	0.070	0.130
PHQ7	0.048	0.000	0.000	0.000	0.040	0.000	0.095	0.000	0.000	0.022
PHQ8	0.136	0.104	0.045	0.066	0.137	0.106	0.000	0.170	0.070	0.003
PHQ9	0.000	0.150	0.062	0.000	0.201	0.127	0.000	0.052	0.256	0.000
Loneliness	0.208	0.108	0.017	0.014	0.021	0.049	0.014	0.025	0.044	0.450

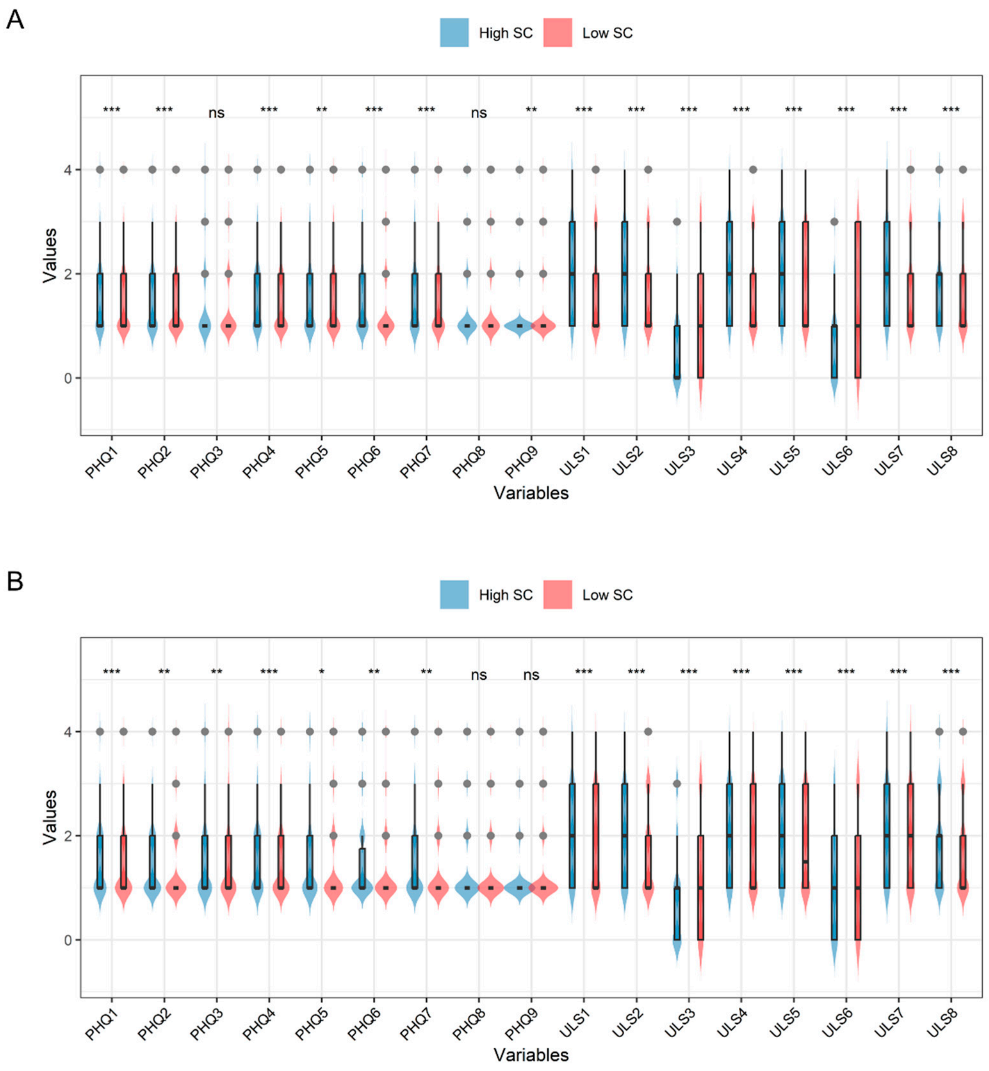

Figure S1. The *t*-test results between two groups at two time points. A. First time point. B. Second time point.

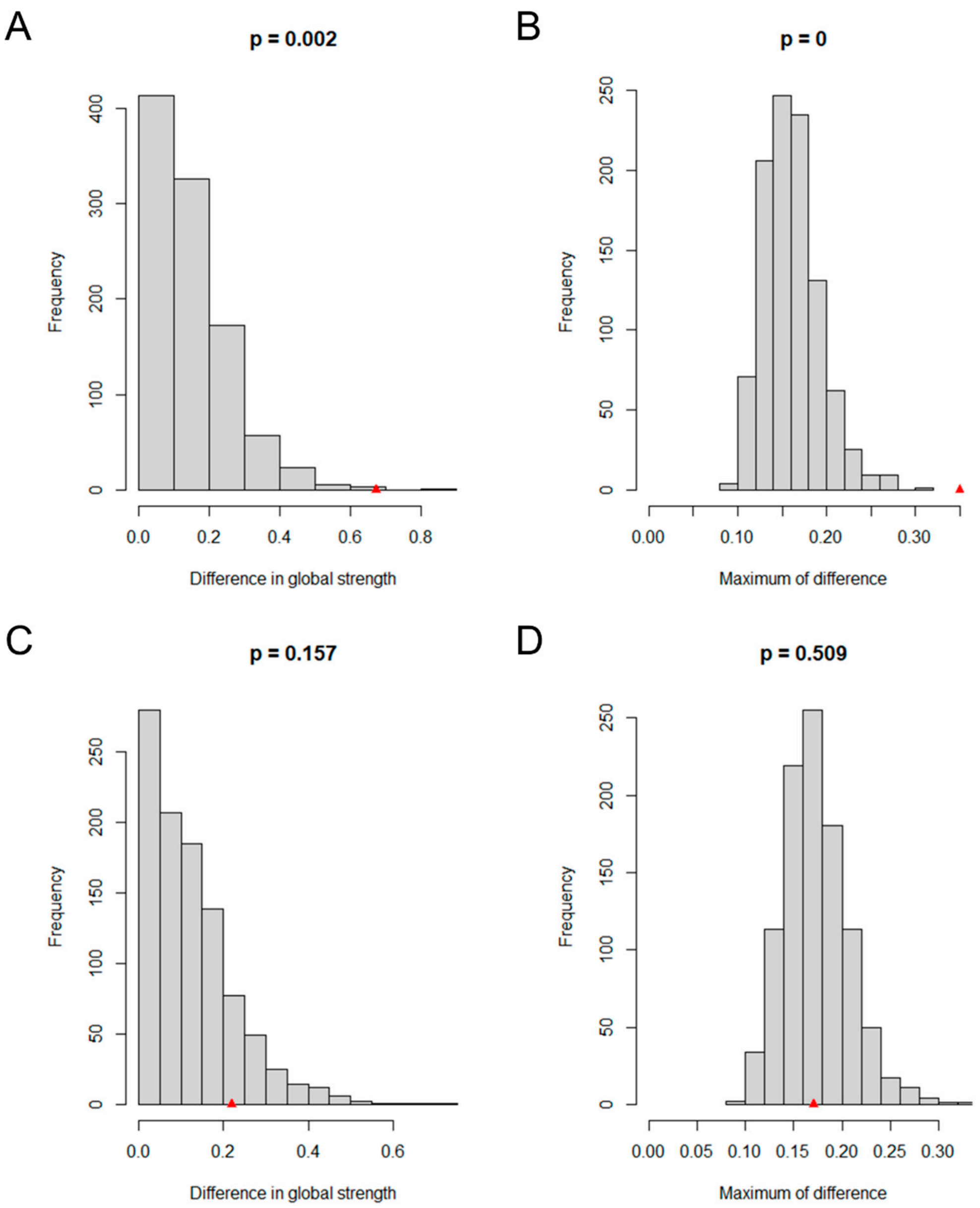

Figure S2. NCT results between two contemporaneous networks. A. Network global invariance test at the first time point. B. Network edge invariance test at the first time point. C. Network global invariance test at the second time point. D. Network edge invariance test at the second time point.

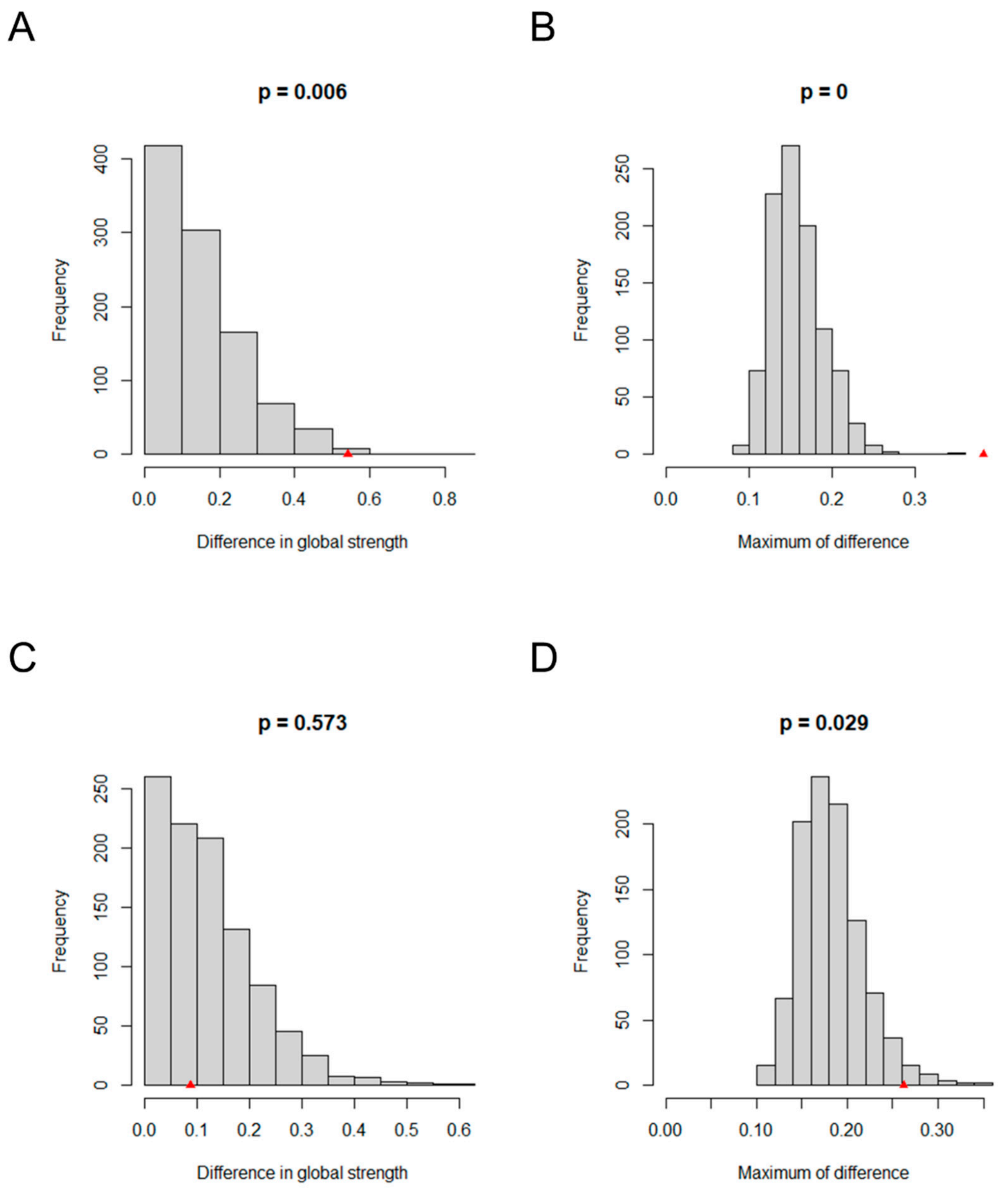

Figure S3. Longitudinal NCT results. A. Network edge invariance test for high-SC group. B. Network global invariance test for high-SC group. C. Network edge invariance test for low-SC group. D. Network global invariance test for low-SC group.

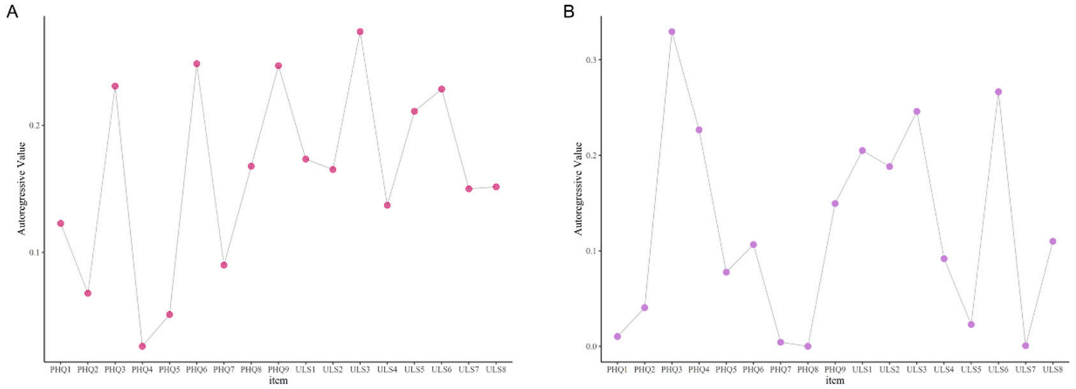

Figure S4. Autoregressive coefficients of each node. A. High-SC group. B. Low-SC group.

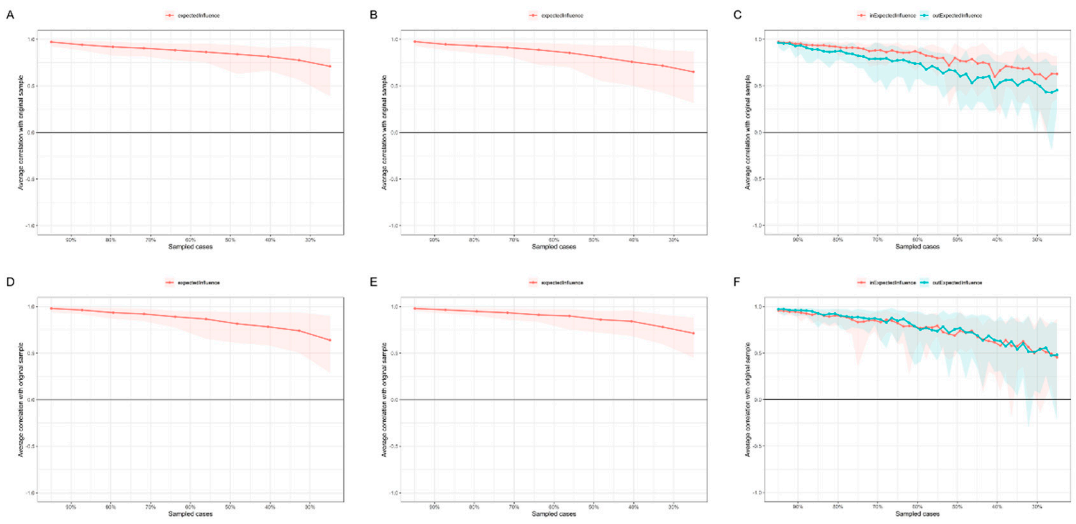

Figure S5. Case-dropping bootstrap test of centrality indices. The x-axis indicates the percentage of cases of the original sample included at each step. The y-axis indicates the correlations between the centrality indices from the original network and the indices from the networks re-estimated after excluding increasing percentages of cases. A. Cross-sectional network of high-SC group at the first time. B. Cross-sectional network of high-SC group at the second time. C. CLPN of high-SC group. D. Cross-sectional network of low-SC group at the first time. E. Cross-sectional network of low-SC group at the second time. F. CLPN of low-SC group.

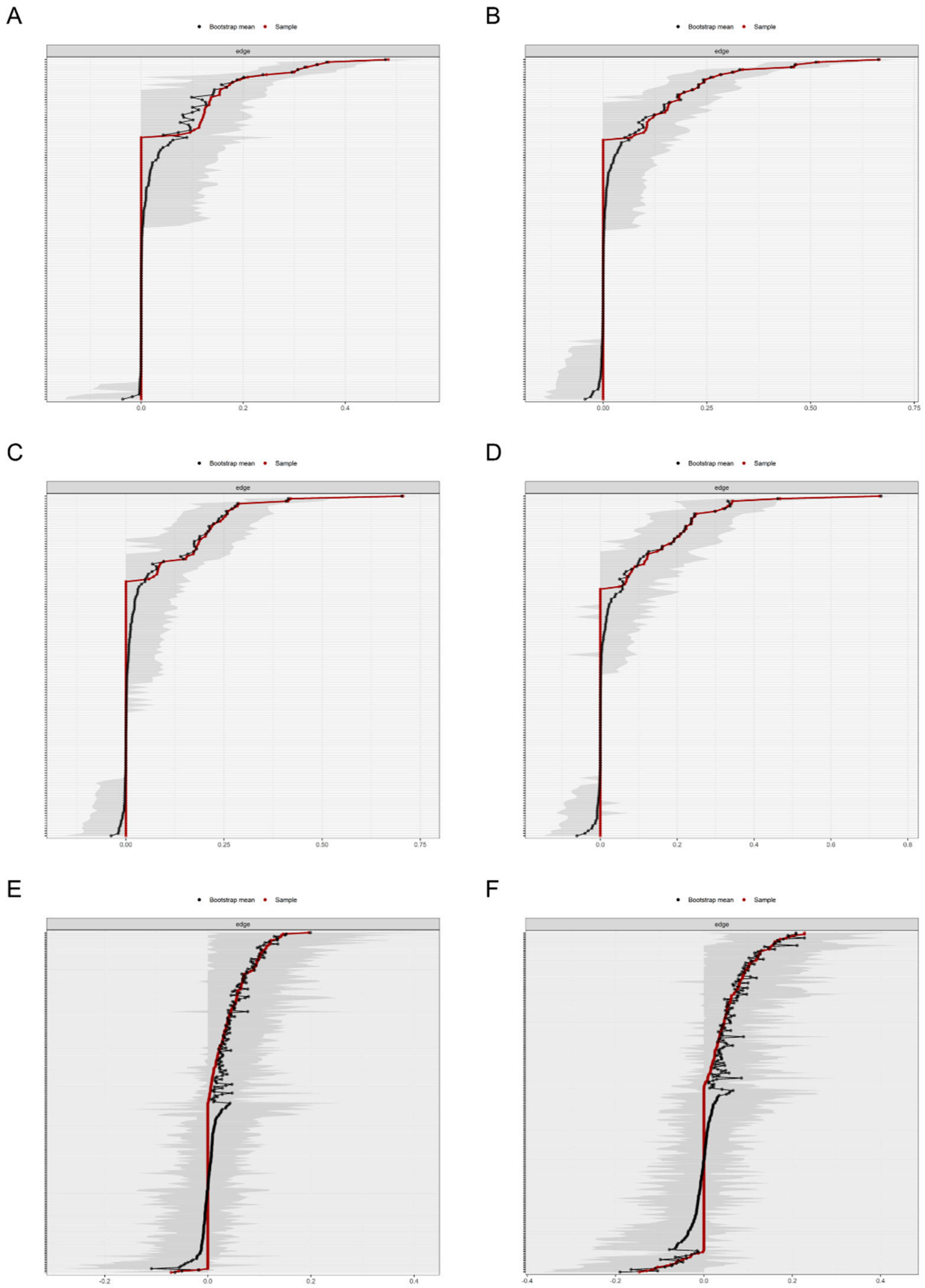

Figure S6. Nonparametric bootstrapped confidence intervals of estimated edges. The red line represents the estimated edge, while the dark area indicates the 95% bootstrap confidence interval. A. High-SC group at the first time point. B. High-SC group at the second time point. C. Low-SC group at the first time point. D. Low-SC group at the second time point. E. CLPN of high-SC group. F. CLPN of low-SC group.

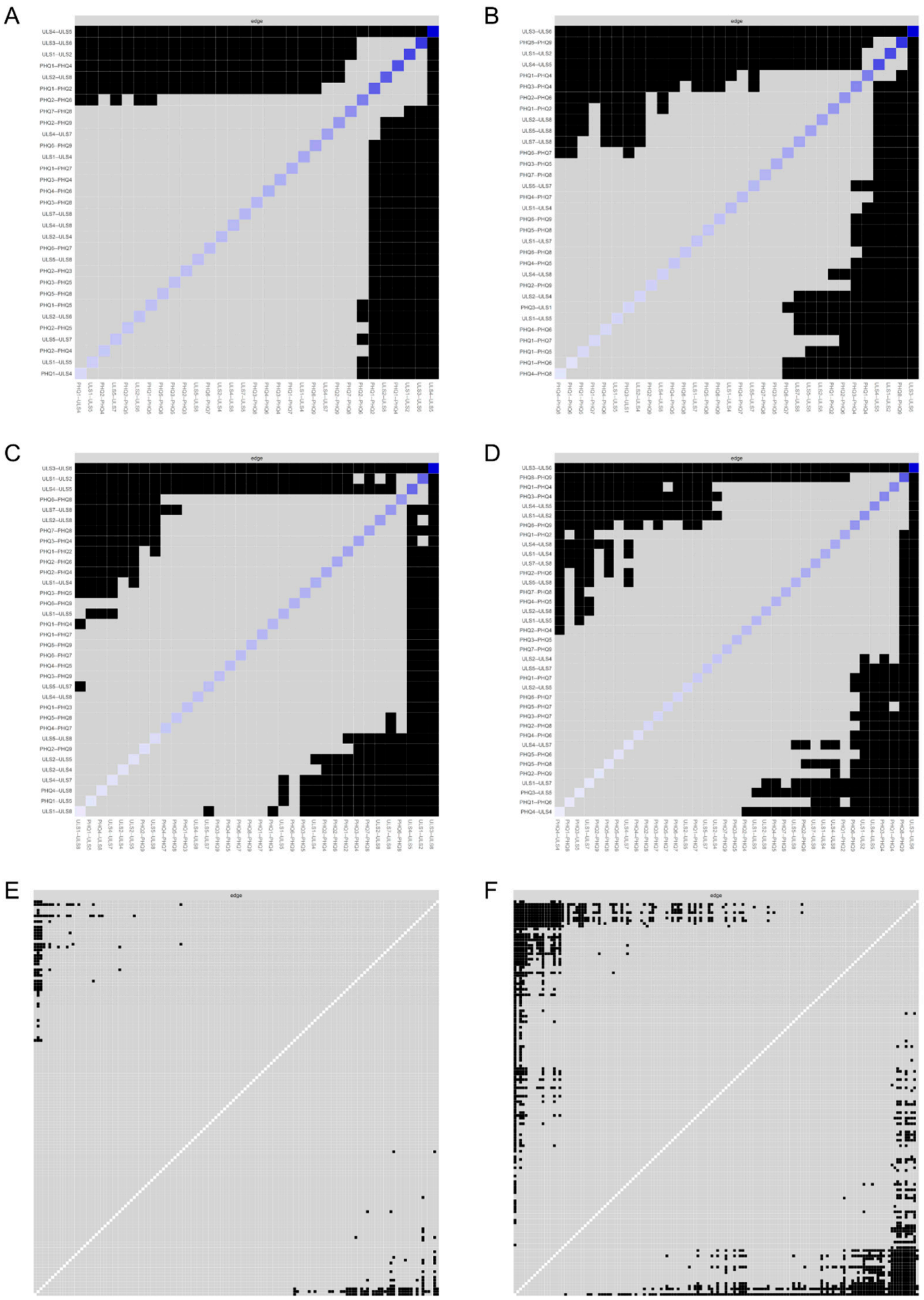

Figure S7. The results of the bootstrapped difference tests ($\alpha = 0.05$) for pairwise edge weights are shown in this figure. The color of the boxes indicates whether edge weights differ significantly from each other (i.e., black) or do not differ significantly (i.e., grey). A. High-SC group at the first time point. B. High-SC group at the second time point. C. Low-SC group at the first time point. D. Low-SC group at the second time point. E. CLPN of high-SC group. F. CLPN of low-SC group.

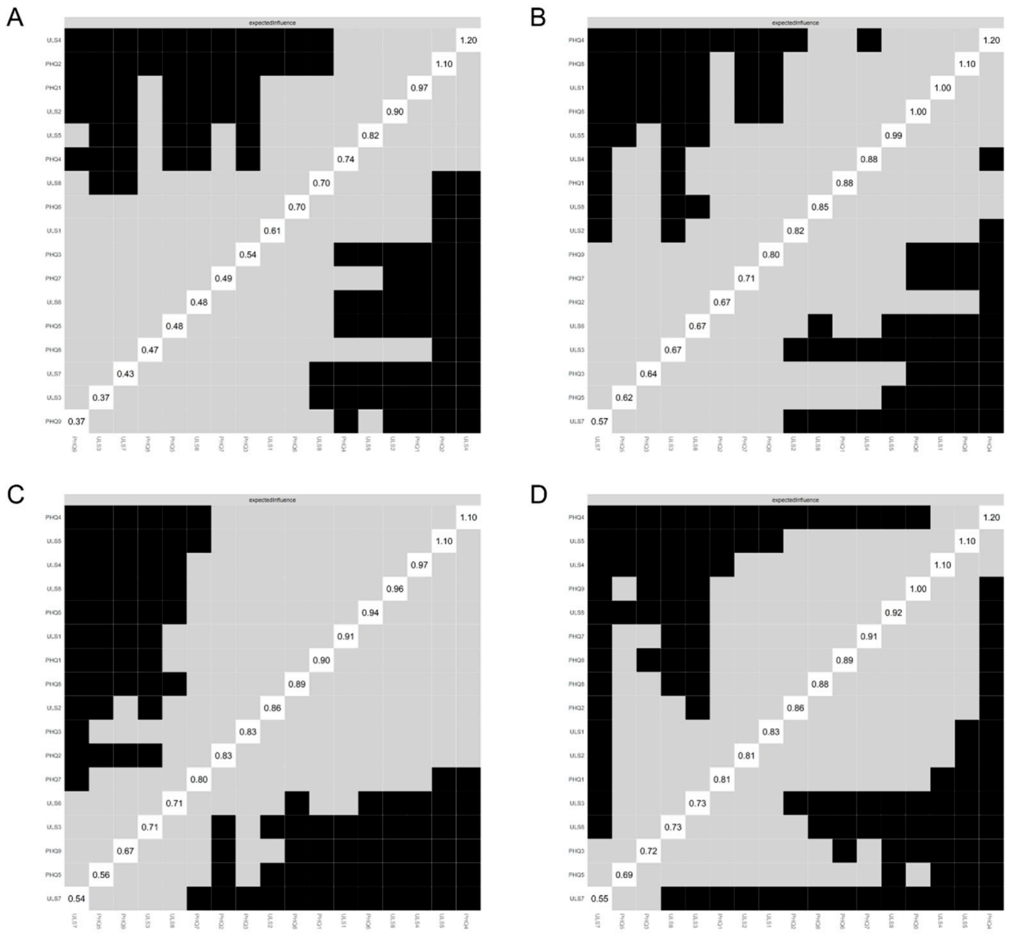

Figure S8. Nonparametric bootstrapped difference test for each node's centrality indices. Grey boxes indicate no significant difference, whereas black boxes indicate a statistically significant difference ($p < 0.05$). A. High-SC group at the first time point. B. High-SC group at the second time point. C. Low-SC group at the first time point. D. Low-SC group at the second time point.

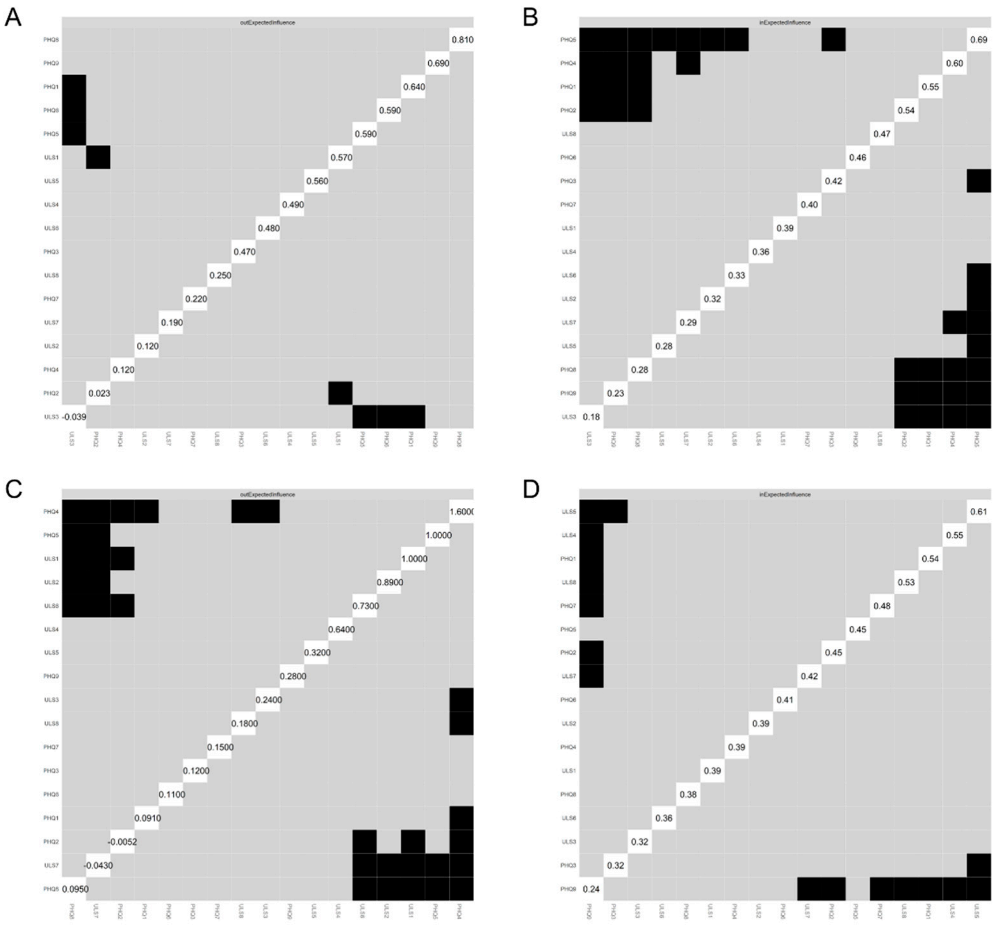

Figure S9. Bootstrapped difference test for each node's *OEI* and *IEI*. A. *OEI* for high-SC group. B. *IEI* for high-SC group. C. *OEI* for low-SC group. D. *IEI* for low-SC group.
